# Ultrasound modulation of macaque prefrontal cortex selectively alters credit assignment–related activity and behavior

**DOI:** 10.1126/sciadv.abg7700

**Published:** 2021-12-15

**Authors:** Davide Folloni, Elsa Fouragnan, Marco K. Wittmann, Lea Roumazeilles, Lev Tankelevitch, Lennart Verhagen, David Attali, Jean-François Aubry, Jerome Sallet, Matthew F. S. Rushworth

**Affiliations:** 1 Wellcome Centre for Integrative Neuroimaging (WIN), Department of Experimental Psychology, Tinsley Building, Mansfield Road, Oxford OX1 3TA, University of Oxford, Oxford, UK.; 2 School of Psychology, University of Plymouth, Plymouth, UK.; 3 Donders Institute for Brain, Cognition and Behaviour, Radboud University Nijmegen, Nijmegen, 6525 HR, Netherlands.; 4 Physics for Medicine Paris, ESPCI Paris, INSERM, CNRS, PSL Research University, Paris, France.; 5 GHU PARIS Psychiatrie and Neurosciences, site Sainte-Anne, Service Hospitalo-Universitaire, Pôle Hospitalo-Universitaire, Paris 15, F-75014 Paris, France.; 6 Université de Paris, F-75005 Paris, France.; 7 Université Lyon 1, Inserm, Stem Cell and Brain Research Institute U1208, 18 Avenue Doyen Lepine, 69500 Bron, France.

## Abstract

Credit assignment is the association of specific instances of reward to the specific events, such as a particular choice, that caused them. Without credit assignment, choice values reflect an approximate estimate of how good the environment was when the choice was made—the global reward state—rather than exactly which outcome the choice caused. Combined transcranial ultrasound stimulation (TUS) and functional magnetic resonance imaging in macaques demonstrate credit assignment–related activity in prefrontal area 47/12o, and when this signal was disrupted with TUS, choice value representations across the brain were impaired. As a consequence, behavior was no longer guided by choice value, and decision-making was poorer. By contrast, global reward state–related activity in the adjacent anterior insula remained intact and determined decision-making after prefrontal disruption.

## Teaser

Noninvasive ultrasound stimulation of primate area 47/12o disrupts credit assignment–related behavior and neural activity.

## INTRODUCTION

**Fig. 1.**
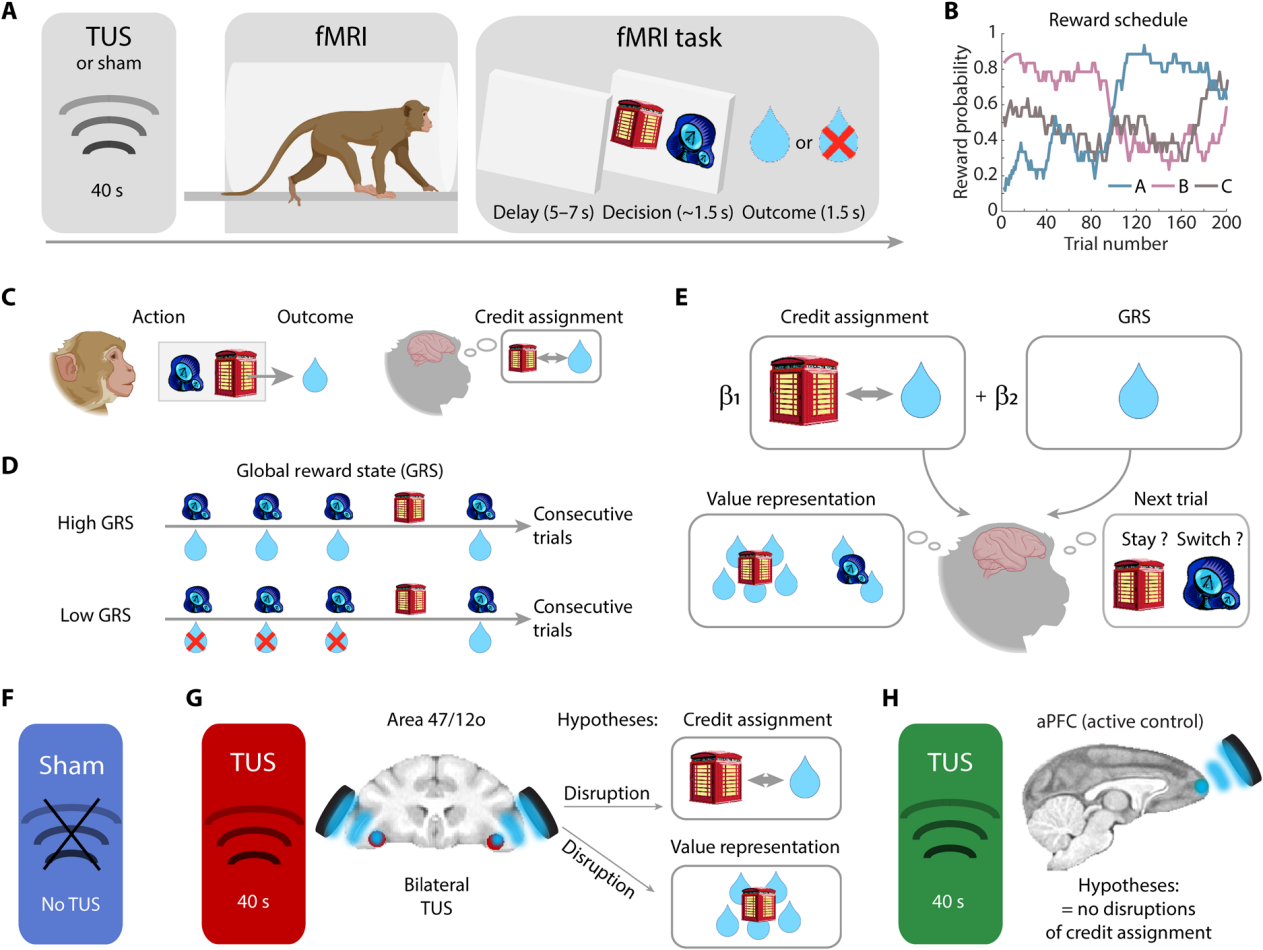
Experimental design. (**A**) Four macaques learned to choose one of the two options represented by visual stimuli presented on a computer monitor by pressing an adjacent touch sensor while in the MRI scanner. The two options presented on each trial were drawn from a larger set of three options. Macaques learned about new stimulus sets in each daily testing session. (**B**) Each option, if chosen, had a probabilistic association with juice reward delivery that drifted over time during the course of the experiment. The reward histories for the three options were uncorrelated with one another (<22% mean shared variance), making it possible to identify neural activity that was correlated with the value of each one of the three options. (**C**) We examined the neural circuits encoding different features of rewards. This includes the specific relationships between choices and outcomes essential for credit assignment and (**D**) the general value of the environment regardless of the specific choice taken, a feature of reward that we refer to as the GRS. (**E**) To test our hypotheses, we also investigated the choice value representations that result from credit assignment and the decision to stay or switch on future trials. (**F**) We used three experimental TUS settings: Sham, where no stimulation was applied; (**G**) TUS targeting a region in area 47/12o, hypothesized to disrupt both credit assignment and value representations that depend on credit assignment; and an active control condition (**H**) in which TUS was applied to anterior prefrontal cortex (aPFC), hypothesized to cause no disruption in credit assignment and value representation. Blue, red, and green color codes indicate results relating to sham, 47/12o, and aPFC conditions, respectively, in the subsequent figures.

In order for behavior to be adaptive, a decision-maker needs to know how good each of its options really is (*1*). While it is important to track how good the environment is in a general sense, it is also essential to know whether the beneficial consequences, such as food rewards that followed a choice, were just a feature of the current environment or whether they were actually caused by the choice. We refer to the process of assigning a particular occurrence of a reward outcome to a particular choice as credit assignment. Here, we examine the neural circuits encoding different features of rewards including (i) the specific relationships between choices and outcomes essential for credit assignment; (ii) the choice value representations that result from credit assignment; and (iii) the general value of the environment regardless of the specific choice taken, a feature of reward that we refer to as the global reward state (GRS). A recent computational model proposed by Wittmann and colleagues (*2*) decomposes the influence of past experience on the next choice taken into a component due to cause-and-effect learning guided by credit assignment and a component due to GRS. By combining this approach with a novel combination of noninvasive transcranial ultrasound stimulation (TUS) (*3*–*5*) and functional magnetic resonance imaging (fMRI) in four macaques performing a behavioral task (Fig. 1, A and B), we recorded activity across the brain during normal credit assignment and when activity in a brain circuit supporting credit assignment was disrupted by TUS.

We focused on the three different reward-related brain signals described above (Fig. 1, C to E): (i) the credit assignment process itself, (ii) the choice value representations that result from credit assignment and that guide decision-making, and (iii) GRS. Credit assignment and the resulting choice value representations were associated with activity in two brain areas—prefrontal cortical area 47/12o, at the border between orbital and ventral prefrontal cortex and anterior cingulate/medial frontal cortex, respectively, while GRS estimates were linked to anterior insula (IA) activity. Application of TUS to 47/12o not only disrupted credit assignment–related activity in 47/12o and adjacent cortex but also disrupted the representation of choice value estimates that result from credit assignment in distant areas, such as anterior cingulate/medial frontal cortex. By contrast, however, the GRS signals, which do not depend on credit assignment, remained in IA despite the spatial proximity of IA to the stimulated 47/12o region. Moreover, after 47/12o TUS, it was this remaining GRS signal that now guided behavior.

## RESULTS

Four macaques learned to choose between two objects presented on a computer by pressing an adjacent touch sensor (Fig. 1, A and B). On each trial, the two objects were drawn from a set of three used during each day’s testing. Each object had a different drifting probabilistic association with reward (Fig. 1B), which was uncorrelated with that of the other objects (<22% mean shared variance). When credit assignment is operating normally, each macaque’s evaluation of a choice should reflect the history of reward experienced in conjunction with that choice; reward experienced after taking a choice should determine that choice’s value and therefore whether it is chosen in subsequent decisions. We used a series of analyses and reinforcement learning models to test whether this was the case.

**Fig. 2.**
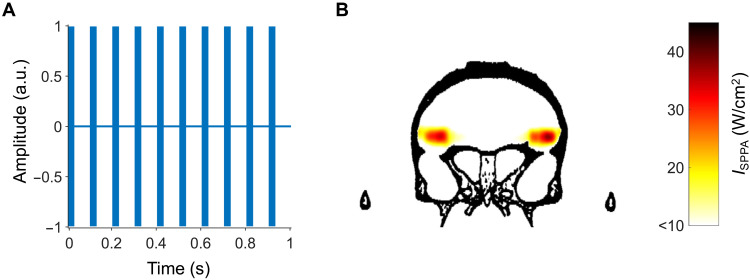
Estimated peak intensities and spatial distributions of ultrasound based on a high-resolution macaque whole-head computed tomography scan. (**A**) Before each session, the TUS protocol consisted of a 40-s ultrasonic stimulation with a rectangular envelope over a train of pulses: The pattern displayed was repeated 40 times. Within the ultrasonic stimulation pulse train, each pulse had a pulse duration of 30 ms and a pulse repetition interval (PRI) of 100 ms for a duty cycle of 30%. The pulse repetition frequency (PRF) within the stimulus duration was 10 Hz. PRF = 1/PRI. a.u., arbitrary units. (**B**) Simulated focused ultrasound peak intensities and spatial distribution in the brain when targeting right and left area 47/12o, based on a high-resolution macaque whole-head computed tomography scan. The maximum spatial-peak pulse-averaged intensity (*I*_SPPA_) at the acoustic focus point was 42.6 W/cm^2^ for the left area 47/12o target and 34.6 W/cm^2^ for the right area 47/12o target. aPFC TUS protocol was the same as the one we previously used in (*8*). Because the beams do not overlap, there is no region of dark red as we have estimated that there has been in some of our previous studies, in which beam overlap was intended (*9*). TUS occurred several minutes before the behavioral task was performed. Such a stimulation protocol is often referred to as an “offline protocol.”

We examined win-stay/lose-shift behavior in no-TUS condition (sham control condition) and after TUS (Fig. 1E). TUS was applied using procedures similar to those previously shown to affect either neural activity or behavior (*3*, *4*, *6*–*10*). In one condition, our main condition of interest, TUS was focused on 47/12o, a region previously linked to win-stay/lose-shift behavior (*11*), situated close to the posterior lateral orbitofrontal sulcus and adjacent to IA. The region’s functional connectivity suggests correspondence with a human brain region, often referred to as lateral orbitofrontal cortex (lOFC) but probably comprising the same 47/12o region, linked to choice-outcome learning (*12*–*15*). Modeling suggested that ultrasound was distributed in an approximately cigar-shaped cylinder where the peak intensity was in 47/12o. The maximum spatial-peak pulse-averaged intensity (*I*_SPPA_) at the acoustic focus point was approximately 35–40 W/cm^2^ in the left and in the right area 47/12o (Fig. 2 and fig. S1). In addition, in another active control condition, we examined the impact of TUS to an anterior prefrontal cortex (aPFC) area that has not previously linked to credit assignment.

It has been argued that it is the acoustic radiation force of the propagating TUS wave that affects neurons (*16*). This stretches the cell membrane so that ion channels are transiently opened (*17*). While active discussion about the mechanism through which TUS exerts its effects continues, what is clear is that it has a relatively circumscribed effect on neural activity but can operate at a distance from the transducer and so it can be used to stimulate deep cortex and subcortical brain areas in a relatively selective manner. TUS has been applied to aPFC, anterior cingulate cortex (ACC), supplementary motor area, basal forebrain, and amygdala, while whole-brain measurements of neural activity were obtained with fMRI. The whole-brain connectome (a measure of the patterns of correlation between each voxel in the brain and everywhere else in the brain) has been estimated and compared with and without TUS. The pattern of interaction between the stimulated region and the rest of brain changes, but patterns of connections between most other brain regions remain unchanged (*6*–*9*). It has also been shown that when it is applied to a brain region, then specific behavioral processes are disrupted (*3*, *4*, *7*, *9*, *18*). The effects are transient, but with some stimulation protocols, they outlast the stimulation period and last for somewhere between half an hour and a day. They occur in the absence of evidence of permanent tissue damage (*8*, *19*). In the current study, we applied a short TUS train for 40 s and, within 10 or 15 min, begun measuring its impact on neural activity and behavior over a subsequent period of several tens of minutes, while macaques performed a task (Fig. 1). Similar TUS protocols have previously been shown to affect either neural activity or behavior (*3*, *4*, *6*–*10*). Unlike in previous studies, which focused either on recording neural activity or behavioral effects in the absence of neural recordings, here, we recorded TUS’s effect on behavior and neural activity throughout the whole brain with fMRI. In this way, we sought to manipulate neural activity, measure the effects of this manipulation in a specific primate brain circuit, and record the impact of this manipulation on behavior.

**Fig. 3.**
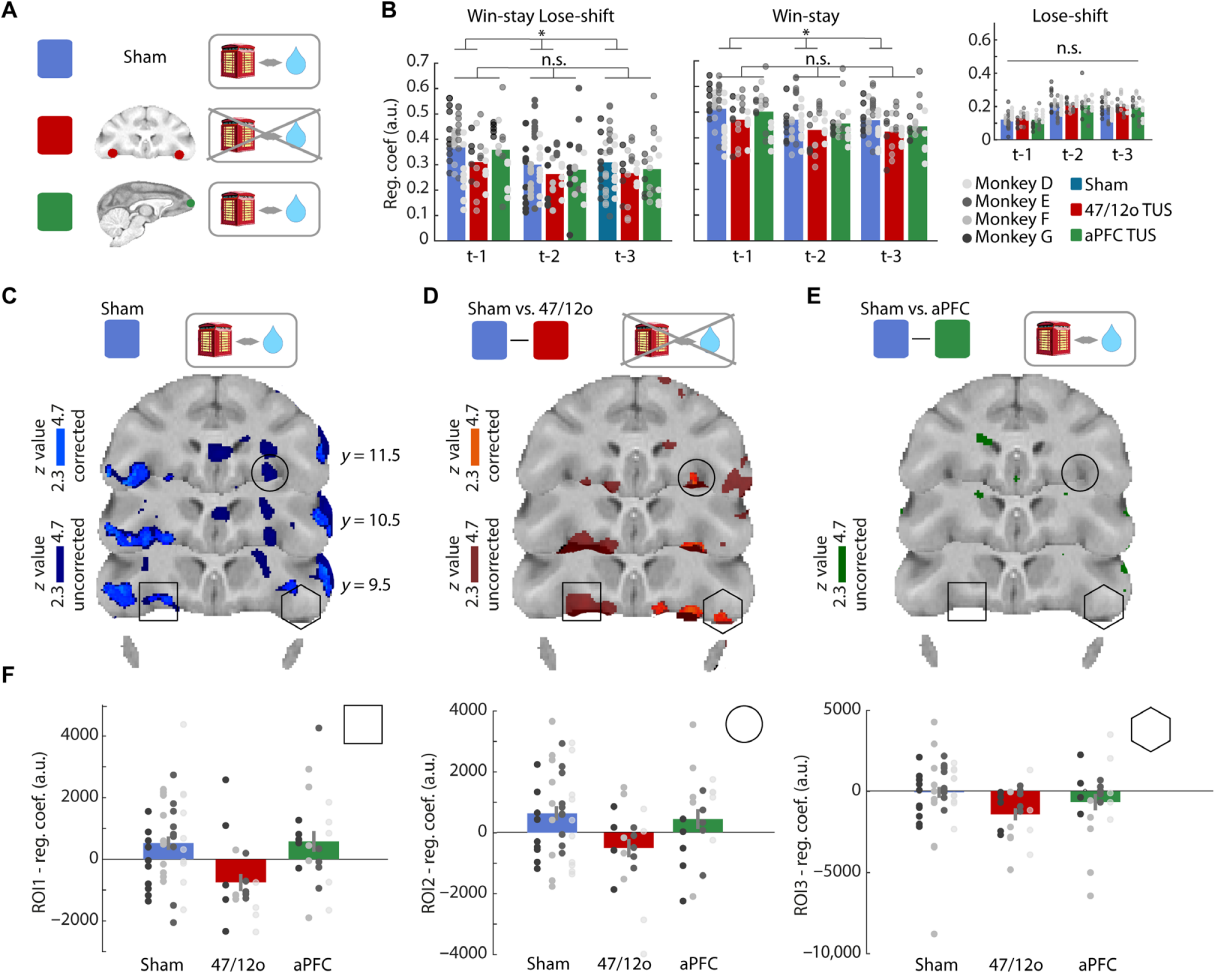
Area 47/12o TUS disrupts adaptive win-stay/lose-shift behavior and neural activity. (**A**) Hypotheses: 47/12o TUS (red) should disrupt credit assignment compared to sham (blue), but aPFC TUS (green) should have no impact. (**B**) Animals exhibited win-stay/lose-shift behavior; they repeated choices after receiving a reward for choosing it previously (win-stay), and they switched away from choices that had previously been unrewarded (lose-shift). Regression coefficients illustrate influence of outcomes on whether win-stay/lose-shift strategy will be taken (left), the win-stay component will be taken (center), or the lose-shift component will be taken (right). Win-stay/lose-shift frequency remained unchanged after aPFC TUS but was significantly reduced after 47/12o TUS. This primarily reflected a decrement in win-stay behavior but not lose-shift behavior (*P < 0.05; n.s., not significant). (**C**) Neural activity underlying adaptive behavior was prominent in sham condition in 47/12o (cluster-corrected, |*z*| > 2.3, *P* < 0.05; light blue) and extended into adjacent orbitofrontal and ventrolateral prefrontal cortex when examined without cluster-based correction for multiple comparison (darker blue). (**D**) However, this pattern was altered by area 47/12o TUS; adaptive behavior–related activity in area 47/12o was significantly reduced compared to sham (comparison of effects estimated from last three occasions choice taken). After cluster-based correction for multiple comparisons, activity was reduced in 47/12o and adjacent orbitofrontal cortex (|*z*| 2.3, *P* < 0.05; light red color indicates significant difference between sham versus 47/12o TUS; dark red indicates activity without cluster-based correction 4.7 < *z* > 2.3; the area of altered activity overlapped with that seen in sham condition in (C). (**E**) No changes in adaptive behavior–related activity were apparent after aPFC TUS versus sham (empty brain indicates no significant difference). (**F**) Illustrated by BOLD effects from 1.5-mm-radius spherical ROIs centered on regions identified by comparison of the adaptive behavior effect in sham and 47/12o TUS condition. No similar changes in adaptive behavior–related activity were apparent after aPFC TUS in these ROIs.

Win-stay/lose-shift (WSLS) behavior provides a direct index of credit assignment that is independent of any particular computational modeling approach because it examines whether a choice’s outcome on one occasion influences whether the choice will be taken on the next occasion that it is available (*11*, *20*, *21*). If a monkey wins a reward for taking a choice, then it should be more likely to repeat that choice on the next occasion that it is available (even if this may only be several trials later in the current paradigm because only two of the three possible options are offered on each trial). By contrast, if the macaque loses, then it should be more likely to shift away to take the alternative choice (*20*). Both win-stay and lose-shift are adaptive strategies indicative of credit assignment. By contrast, the other possible behavioral strategies, win-shift and lose-stay, are maladaptive ones. Simple win-stay/lose-shift strategies are especially adaptive in deterministic tasks, in which one choice is always rewarded and the other option is never rewarded. Reward is, however, probabilistically associated with choice in the current experiment so, rather than just a simple win-stay/lose-shift strategy that relates the last outcome to the next choice, each outcome should have some impact not only on the next choice taken but also on choices even in later trials. That is, each choice should be influenced by outcomes not only on the previous trial but also across several previous trials. We therefore considered the influence that outcome for a choice had on the next occasion (t-1), the subsequent occasion (t-2), and the occasion after that (t-3) on which the same choice was presented. Because only two of the three options were presented on each trial, a choice might not be available over several consecutive trials, and so, this analysis approach meant that we frequently examined the impact each outcome had for more than three trials in the future. We considered the combined impact that winning had on staying and losing had on switching (Fig. 3B, left), the impact wins had on staying (Fig. 3B, center), and the impact losses had on switching (Fig. 3B, right). In each case, we used a logistic regression approach so that the impacts of outcomes on subsequent behavior are reported as regression coefficients rather than simply as frequencies of win-stay/lose-shift, win-stay, or lose-shift (Fig. 3B).

Analyses were conducted with a linear mixed-effects model that included condition (either sham versus 47/12o TUS or sham versus aPFC TUS) and trial history (looking at the impact that outcomes on the current trial had for behavior one, two, or three trials into the future) as fixed effects and subjects as random effects. In the sham control condition, all four macaques exhibited win-stay/lose-shift behavior (Figs.1, F to H, and 3, A and B). However, 47/12o TUS disrupted crediting positive outcomes, reward, to the choice made [win-stay strategy: *t*_(177)_ = −4.19, *P* < 0.0001; fig. S2] but not lose-shift strategy compared to sham [*t*_(177)_ = 0.66, *P* = 0.51; Fig. 2B and fig. S2]. By contrast, aPFC TUS was no different to sham [win-stay: *t*_(177)_ = −1.61, *P* = 0.11; lose-shift: *t*_(177)_ = 0.07, *P* = 0.95; Fig. 3B and fig. S2].

A similar general linear model (*GLM1*) sought fMRI activity associated with adaptive behavioral strategies throughout the brain; GLM1 identified fMRI activity associated with adaptive win-stay/lose-shift strategies after controlling for the influence of maladaptive win-shift/lose-stay behaviors. The fMRI analysis was conducted in an analogous manner to the behavioral analysis and considered the impact of outcomes on the current trial on stay/shift behaviors over the next three occasions on which each option was offered (see the “GLM1—Neural activity related to adaptive behavioral strategies” under “Whole-brain GLMs” section; Fig. 3B and fig. S2). In the sham condition, activity in orbitofrontal cortex centered on 47/12o (in and adjacent to the lateral orbital sulcus) encoded adaptive behavioral strategies following both positive (win-stay) and negative outcomes (lose-shift) (Fig. 3C, blue) (*11*). The pattern of activity associated with the adaptive win-stay/lose-shift behavior was, however, significantly reduced after 47/12o TUS (Fig. 3D, red, and fig. S2), although it remained unchanged after control aPFC TUS [Fig. 3E, green, and fig. S2, and see for illustration regions of interest (ROIs) 1 to 3 in 47/12o].

**Fig. 4.**
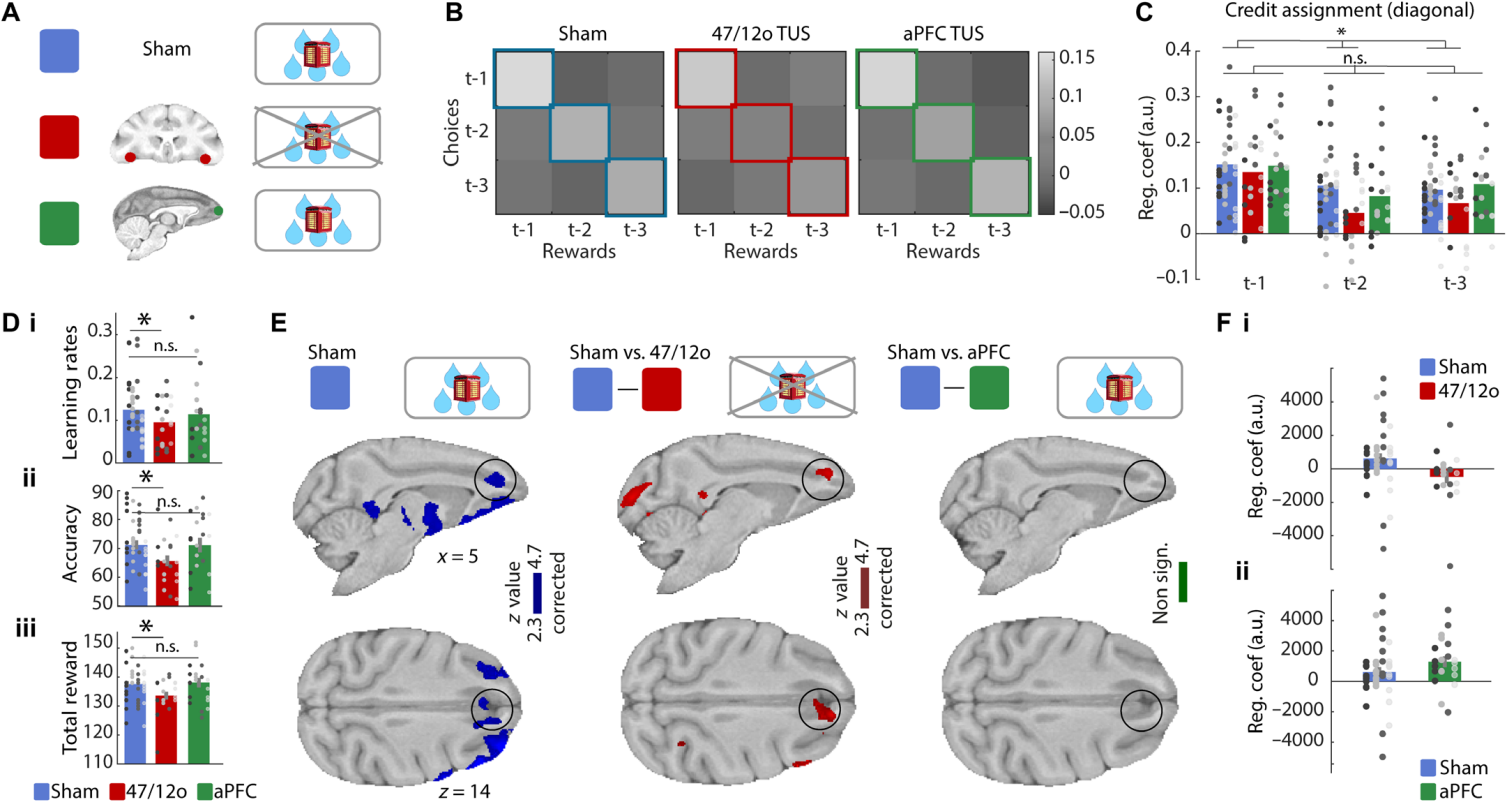
Area 47/12o TUS disrupts credit assignment and reduces choice value representation in ACC. (**A**) Hypotheses: 47/12o TUS (red) should disrupt value representations used for decision-making that are dependent on credit assignment compared to sham (blue) and aPFC TUS (green). (**B**) The influence, on which choice is taken next, of past reward history (abscissa), choice history (ordinate), and their conjunction (main diagonal) in sham (left), after 47/12o TUS (middle), and aPFC TUS (right). Labels t-1, t-2, and t-3 refer, respectively, to the last occasion, the previous occasion, and the occasion before that, on which a given stimulus was encountered. In sham, choices are influenced by the conjoint history of choice and reward (regression weights on main diagonal are high). (**C**) The influence of choice-reward conjunctions [regression weights from diagonal in (B)] in all three conditions is also replotted as a histogram. It was significantly reduced after 47/12o TUS but not aPFC TUS (*P < 0.05; n.s., not significant). (**D**) (i) The difference in choice value learning after 47/12o TUS was also attested by lower learning rates compared to sham and aPFC TUS. (ii) Area 47/12o TUS reduced the frequency with which macaques took the option estimated by the reinforcement learning model to have the highest value in comparison to sham and aPFC TUS and (iii) so animals earned fewer rewards. (**E**) In sham, activity in several frontal cortical areas including ACC reflected values of choices that could be taken (cluster-corrected, |*z*| > 2.3, *P* < 0.05). This value signal in ACC was significantly reduced after 47/12o TUS (cluster-corrected, |*z*| > 2.3, *P* < 0.05) but not after aPFC TUS. (**F**) (i and ii) This is illustrated by extracting BOLD effects from 1.5-mm-radius ROIs [black circle (E)]. Separate panels illustrate contrasts of sham and 47/12o TUS (Fi) and sham and aPFC TUS (Fii) on ACC value estimates (models fitted separately for each comparison).

So far, the analyses have focused on the process of credit assignment as indexed by an analysis of win-stay/lose-shift behavior. The credit assignment process determines the choice value estimates that, in turn, guide the monkeys’ decisions (Figs. 1E and 3A). In the next analyses, we focus on these choice value estimates themselves and the way in which they reflect the extended history of choice-reward conjunctions, and we examine how they change when credit assignment is altered by 47/12o TUS (Figs. 1E and 4, A to C) (*22*, *23*).

We investigated how macaques estimated the values of their choices; first, we used an approach independent of any formal, computational model: a logistic regression analysis to predict the next choice that would be made by each macaque as a function of previous choices, previous rewards, or their conjunction (*22*). The brightness of squares in the grid (Fig. 4B) indicates the size of influence of these factors as indexed by the regression coefficients in sham, 47/12o TUS, and aPFC TUS conditions, respectively. The bright diagonal indicates that it is the conjunction of choices made on each recent occasion they were made (t-1, t-2, and t-3) and the rewards received on the same occasions, rather than just the history of choices regardless of reward, or reward receipt regardless of choice, that has the greatest influence on what decision will be made next. The choice-reward history (the diagonal in Fig. 4B) is replotted as a histogram in Fig. 4C. Note that, unlike in behavioral paradigms in which all choices are offered on every trial (*22*, *23*), in the present task, a given option is only offered on a subset of trials, and so t-1, t-2, and t-3 effects indicate influences extending across many trials in the past. While there was no difference in past choice-reward conjunction effects after sham and control aPFC TUS [*t*_(177)_ = −0.4, *P* = 0.69; Fig. 4B], there was a significant difference between sham and 47/12o TUS [*t*_(177)_ = −3.06, *P* < 0.01; Fig. 4B].

**Fig. 5.**
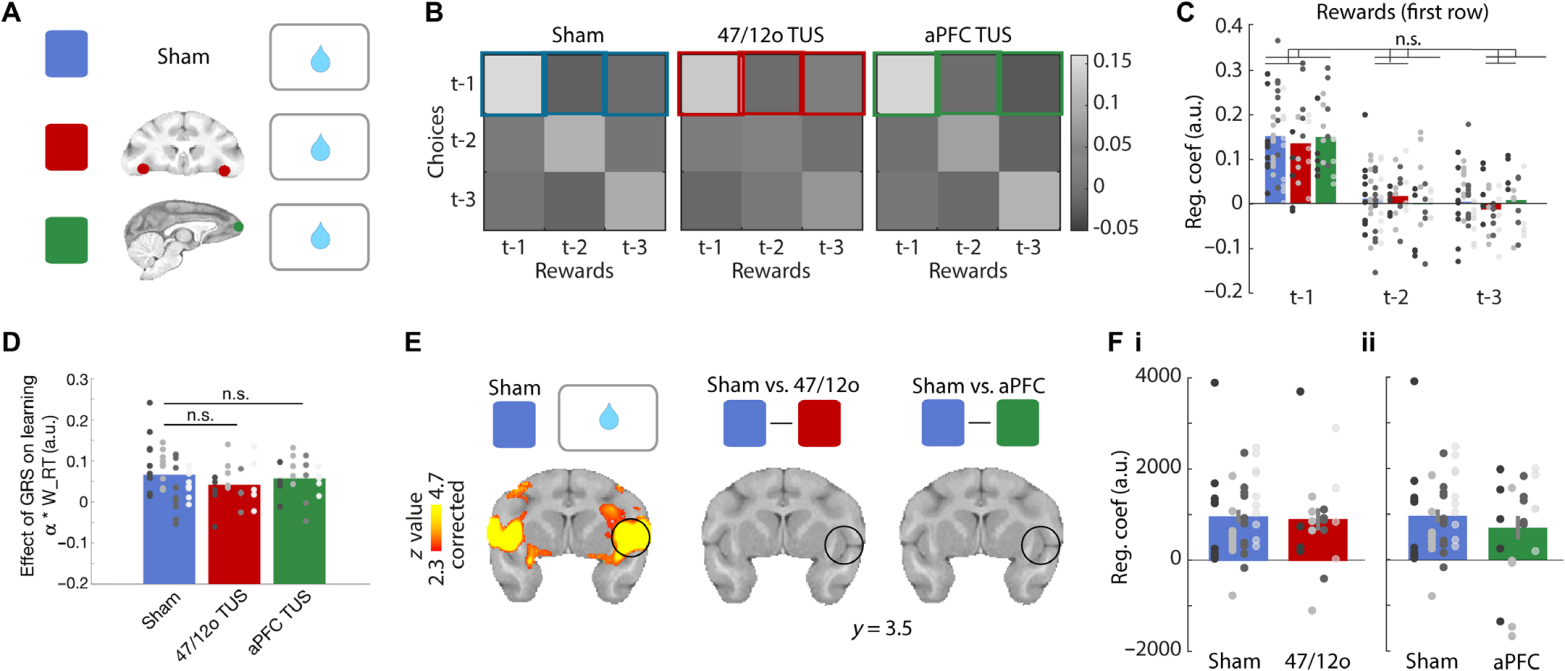
Area 47/12o TUS does not alter GRS impact on behavior or neural activity. (**A**) Hypotheses: 47/12o TUS (red) should not disrupt GRS representations (illustrated as in Fig. 1E). In addition, no effect of aPFC TUS (green) is expected in comparison to the sham control (blue). (**B**) The influence of the past reward history (abscissa, highlighted) on which choice the animal will take next in the sham condition (blue), after 47/12o TUS, and after aPFC TUS. (**C**) The same analysis is visualized as a histogram. (**D**) The overall influence that GRS has on learning was not different across experimental conditions. This was demonstrated by examining W_RT [the weight exerted by GRS when prediction error (PE) learning occurred]. When W_RT is multiplied by the learning rate (how much animals learn on each trial), then this reveals the overall influence that GRS has on learning and this is what is plotted here. (**E**) In sham, parametric variation in GRS was associated with variation in neural activity amygdala, operculum but most prominently IA. There was no change in this activity after either 47/12o TUS or aPFC TUS. (**F**) Effects extracted from IA region shown in inset are shown for comparison of sham and 47/12o TUS and comparison of sham and aPFC TUS. As in Fig. 3, separate panels illustrate contrast of sham and 47/12o TUS (i) and sham and aPFC TUS (ii) because small differences in model fitting resulted in small differences in effect size estimates in the sham group depending on the comparison group when fitting was done (however, careful fitting to either sham versus 47/12o or sham versus aPFC guarded against the possibility that any changes in activity that might have been found could have been a consequence of a poor model fit when TUS was applied).

Next, we used Wittmann’s reinforcement learning model (*2*) to decompose this influence of past experience on future decisions into two components in addition to the choice-specific value estimates based on credit assignment over recent trials [note that this model fits the behavioral data better than a classical reinforcement learning (RL) model (*2*)]. The first component was the GRS, the general value of the environment regardless of the specific choice taken (we return to this below and in Fig. 5D), and the second component comprised choice-specific effects regardless of whether rewards were received; that is, does an animal tend to take the same choice again just because it was taken in the past regardless of whether it was rewarded? With this model, it is possible to estimate how animal learn choice values on each trial (given each individual’s experience of the choices up to that point); if 47/12o TUS disrupts how animal learn choice values, then this should be reflected in a difference in the model’s learning rate parameter in sham and 47/12o conditions because this reflects how past experience shapes choice-specific values [note that although we tested whether there was evidence of different learning rates for positive and negative outcomes, as in previous studies (*2*, *37*), we found that a single learning rate provided the best account of the data; fig. S3]. Second, if choice-specific value representations are compromised, then they may influence decisions less; decisions will become inaccurate. Third, ultimately, animals will obtain fewer rewards. Our results confirmed all three hypotheses. After 47/12o TUS, animals (i) learned choice values in a different way, and this is attested by lower learning rates after 47/12o TUS [47/12o TUS versus sham (sham 47/12o and sham aPFC): *t*_(58)_ = −1.99, *P* = 0.05; 47/12o TUS versus sham 47/12o: *t*_(38)_ = −2.27, *P* = 0.027; Fig. 4Di]; (ii) the value estimates are not used so effectively in decision-making; the choices are less accurate even when one takes into account that they have been learned differently [47/12o TUS versus sham: *t*_(58)_ = −2.27, *P* = 0.027; Fig. 4Dii; aPFC versus sham: *t*_(58)_ = −0.04, *P* = 0.97; fig. S4 illustrates a rolling average of the frequency of correct choices—choices of the highest value throughout the session]; (iii) as a consequence, animals obtained fewer rewards in each session [*t*_(58)_ = −2.39, *P* = 0.02; Fig. 4Diii], but this was not the case when aPFC TUS and sham conditions were compared [*t*_(58)_ = 0.31, *P* = 0.76]. Note that while the first two findings relate to Wittmann’s model, this final measure is independent of the model.

The model’s estimates of choice values on each trial were also used in an analysis of neural activity; the fMRI signal throughout the brain was regressed onto the model’s value estimates using *GLM2*. Our new analysis investigated activity covarying with the values of all options. In previous studies, we have used more specific analyses to capture activity related to the comparison of the value an option that will be chosen and become the focus of attention as opposed to the option that will be rejected (*24*), the activity associated with the value of an option unpresented on the current trial but held in memory, or the activity associated with the best alternative to the current course of action (*7*). Here, the intention is to perform a more general analysis and to capture activity related to the value of any choice and, so, that is why we sought activity covarying with the values of all options [all three object values (*25*, *26*)] on each trial (the option chosen, the option unchosen, and the option that was unpresented but held in memory). Note also that this analysis (Fig. 4E) should identify the neural correlates of choice values, used to guide decisions, that have been learned as a result of credit assignment rather than the credit assignment process itself [which, by contrast, was examined in the win-stay/lose-shift analyses in Fig. 3 (C to E)]. However, because they depend on credit assignment for their construction, these choice value estimates, even if held in a brain area distant from 47/12o, should still be compromised by 47/12o TUS. In the sham condition, mean choice value–related activity (regardless of whether the choice was taken, rejected, or simply unavailable on a given trial) was associated with activity in several prefrontal locations including orbitofrontal and ventrolateral cortex, thalamus, and ACC (Fig. 4E). The activity was reduced after 47/12o TUS and the effect reached statistical significance in ACC (Fig. 4E, center), a region in which activity has been closely linked to representation of choice values and guidance of choice selection (*7*, *9*, *27*–*29*). No such effects were seen after aPFC TUS (Fig. 4E, right). Thus, not only was behavior less guided by choice values derived from specific choice-outcome credit assignment after 47/12o TUS (Fig. 4, B and C), but also, in parallel, ACC was less driven by choice values derived from specific choice-outcome credit assignment after 47/12o TUS (Fig. 4, E to G). Anterior cingulate activity guides monkeys’ value-based decision-making in many circumstances (*7*, *27*–*29*). Previous work has suggested that it reflects the values of alternative choices that the monkey might shift to in the future (*7*), and further analysis confirmed that it was precisely these representations—representations of the values of potential alternatives to the current choice in ACC— that were compromised by 47/12o TUS (fig. S5). While orbitofrontal and ACC, on the other hand, comprise largely separate neural networks in the macaque, area 47/12o is one of the few connection nodes shared between networks (*30*).

The picture of results built up so far has emphasized distinct roles for 47/12o and ACC and noted the presence of activity related to updating of values in WSLS-related analyses in 47/12o and holding of potential choice values to guide decision repetitions or changes in ACC. We examined this account more formally by comparing the relative strengths of value-related activity in or adjacent to 47/12o in the lateral orbital sulcus (square and circle ROIs in Fig. 3D and figs. S6 and S7A) and ACC (ROI in Fig. 4E) using a GLM with factors of brain area (ACC versus 47/12o) and activity type [average value estimated from Wittmann’s (*2*) reinforcement learning model or WSLS-derived updating-related activity]. When we examined the sham control data, we found a significant interaction between the two factors [circle ROI, left hemisphere: *t*_(156)_ = 2.128, *P* = 0.034; square ROI, right hemisphere: *t*_(156)_ = 2.352, *P* = 0.019; figs. S8 and S9] consistent with the WSLS signal being stronger in 47/12o and the value signal being stronger in ACC. Further analysis confirmed that the WSLS was significantly stronger in 47/12o than in ACC [circle ROI, left hemisphere: *t*_(78)_ = −2.35, *P* = 0.0209; square ROI, right hemisphere: *t*_(78)_ = −1.81, *P* = 0.074], although the difference in strengths of value signals in ACC and 47/12o was not significant. This is consistent with the previous observation that the average choice value signal was widely distributed and present in many areas.

When we compared the average value and WSLS signals in 47/12o in the 47/12o TUS, sham control, and aPFC TUS conditions (figs. S8 and S9), we found that, while the WSLS signal was significantly diminished by 47/12o TUS [circle ROI, left hemisphere: *t*_(78)_ = −2.35, *P* = 0.0209; square ROI, right hemisphere: *t*_(78)_ = −3.64, *P* = 0.0004], the average choice value signal did not change across conditions (no significant difference across 47/12o TUS, sham control, and aPFC TUS conditions). A similar pattern (fig. S10) was apparent in the more lateral 47/12o ROI (hexagon in Fig. 3, C to E). Further analyses confirmed that the average value signal (for the chosen, unchosen and unpresented option) did not change after TUS was applied to the 47/12o, a result that differs from the corresponding results for the ACC, confirming the distinct roles of these two regions.

However, despite the impact of 47/12o TUS on credit assignment, the impact of GRS on choices (Figs. 1, D and E, and 5A) was not affected by 47/12o TUS [*t*_(77)_ = −1.47; *P* = 0.144; Fig. 5, B to D]; when 47/12o activity was disrupted, behavior was still guided by a general sense of how good the environment had been recently regardless of the specific choice taken (Fig. 5D; animals carried on making the same choices as previously when GRS was high but switched when it was low). In a final stage of our analysis, we also sought neural activity related to GRS. In the sham condition, it was especially prominent in IA [Fig. 5, E (left) and F (left)]. Despite spatial proximity to IA, 47/12o TUS had no impact on GRS-related activity in IA [Fig. 5, E (center) and F (left)]. In addition, aPFC TUS (active stimulation control condition) had no impact on GRS-related activity in IA [Fig. 5, E (right) and F (right)]. In summary, 47/12o TUS disrupts credit assignment–related processes (Fig. 3), and it weakens choice value representations dependent on this credit assignment process even when they lie at some distance from 47/12o (Fig. 4). However, 47/12o TUS had no effect on the spatially adjacent, but distinct, value representation, GRS (the average value of the recent environment regardless of the specific choices taken), or the behavioral impact of GRS (Fig. 5). The effects of 47/12o are thus specific to 47/12o and to computational processes dependent on 47/12o.

## DISCUSSION

Rewards are primary drivers of behavior. Here, we have focused on two distinct reward-related computations and their impact on behavior: reward credit assignment and GRS associated with neural activity in 47/12o and IA, respectively. By combining noninvasive ultrasound stimulation and computational model–informed whole-brain imaging in macaque monkeys, we were able not only to establish the existence of these circuits but also to demonstrate both a circumscribed impact on activity on the stimulated region and a wider impact on the neural signals that depended on that region using both simple behavioral measurements (win-stay/lose-shift-related analyses, Fig. 3; total rewards received, Fig. 4D) and reinforcement learning model–based analyses (Fig. 4, E to J). Activity related to GRS—the recent history of reward regardless of the specific choices taken—was found bilaterally in IA and was unaffected by 47/12o TUS (despite IA’s proximity to the 47/12o target region). The 47/12o region lies within the larger region identified with credit assignment by Rudebeck and colleagues (*23*). The WSLS activity that we found was present in 47/12o lateral to the lateral orbital sulcus and in the lateral orbital sulcus itself. However, it was likely that it extended into adjacent area 13 l in orbitofrontal cortex and 12 l in the ventrolateral prefrontal cortex. It is possible that other parts of area 47/12 beyond just 47/12o, which extends onto the ventrolateral prefrontal surface, make additional contributions to credit assignment and attention-related processes (*31*, *32*). The combination of approaches used here allowed us to tease apart the functions of areas on the orbitofrontal surface and in cingulate cortex that can appear similar in other investigations (*33*) and furnished a formal description of the different choice-outcome specific processes in these regions and context learning processes in IA (*34*). These results may also have implications for understanding clinical conditions characterized by alterations in learning and decision-making. More generally, the techniques used here add to the range of tools for understanding circuit changes in primate models of brain function (*35*, *36*), but the noninvasive nature of TUS also suggests the possibility of translation of the approach to humans including patients.

## MATERIALS AND METHODS

### Subjects

Four male rhesus monkeys (*Macaca mulatta*), here referred to as D, E, F, and G, were tested in the experiment (mean weight, 12.65 kg; 9.9 years of age). They were socially housed (group of four animals) and kept on a 12-hour light/dark cycle, with access to water for 12 to 16 hours on testing days and with ad libitum water otherwise. Before task training, the animals underwent aseptic surgery to implant an MRI-compatible head post (Rogue Research). The head post was necessary to prevent head movement during the TUS and imaging data acquisition. All procedures were conducted under licenses from the U.K. Home Office in accordance with the U.K. Animals (Scientific Procedures) Act 1986 and with the European Union guidelines (EU Directive 2010/63/EU).

### Behavioral training

All four animals were initially trained to sit in a MRI-compatible chair in a sphinx position and perform a computer-based probabilistic reward-guided reversal learning task while inside a custom-made mock scanner, while mock MRI noise was played in the background for the full length of the session. These mock conditions were implemented to simulate the MRI scanning environment and train the animals to tolerate the testing conditions. Similarly, before each training sessions, the animals were tested in a mock TUS session in which no ultrasonic waves were transmitted through the skull. All animals were trained to reach two custom-made infrared touch sensors (one located in front of the left hand and one located in front of the right hand) to respond to abstract stimuli presented on the left and right of a black screen located in front of the animal. Touching the left and right sensors effected the choices of the left- and right-hand options, respectively. Once the animals reached a learning criterion, the animals were moved to the actual MRI scanner where they performed the task with head fixation at over 70% accuracy. MRI data acquisition started once animals performed above 70% accuracy for more than three consecutive times in the MRI scanner environment.

### Experimental task

Testing was conducted in a horizontal bore 3T MRI scanner with the animals head fixed to the MRI-compatible chair. Animals were required to learn the continually changing reward probabilities associated with three choice options and to adapt their behavior to maximize the amount of reward received. On each trial, two of the three possible stimuli were presented. Animals had to learn the reward probability associated with each choice option by choosing between the two options presented on the screen at each trial and monitoring whether a reward was received for making the choice. The three stimuli used to represent the three options were novel in each testing session (Fig. 1), and each testing session took place on a different day. The task consisted of a probabilistic reward-guided reversal learning task inspired by tasks originally designed to study reinforcement learning (*22*, *37*, *38*). Choice options were allocated pseudo-randomly to the right- and left-hand sides of the screen and the animals responded by reaching a right or left infrared sensor placed in front of each of their hands. The rewards were delivered probabilistically, and the probabilities associated with the three options fluctuated throughout the session, with the probability of two of the options reversing toward the middle of a session (Fig. 1). At each time point during the task, animal faced one “high probability option” and two “low probability options.” When deciding between the two low probability options, animals chose the one that carried the highest expected value as estimated on the basis of past reward history. This manipulation ensured a more balanced number of positive and negative outcomes.

To control for reward schedule-induced biases in the animals’ choice preferences, two macaques were presented with one reward schedule and the other two with a second reward schedule with matching reward rates for the three stimuli. Thus, in both reward schedules, the probability range for option A was 90 to 10%, the probability range for option B was 70 to 30%, and the probability range for option C was 10 to 90%.

As mentioned above, although three choice options were presented in each session, only two of them could be chosen on each trial (Fig. 1). This manipulation requires the animals to learn the contingency between the selected choice option and the subsequent outcome. Thus, to maximize the amount of reward received, the animals are required to use these choice option-outcome associations to guide behavior. Moreover, the probabilistic nature of the task required animals to continually monitor the outcomes that followed choices of each option chosen. For instance, in a given trial, the macaque may have chosen the stimulus associated with the highest likelihood of reward but may not have been rewarded for making that choice. The choice’s valuation should reflect not only what happened on that trial but also whether outcomes were delivered recently when the same choice was taken. The animals had also to keep track of outcomes received in previous trials because only two of the three options were presented on any given trial. This task is, therefore, ideal for investigating the capacity of animals to integrate the past history of choice option-outcome conjunctions to guide future behavior. Furthermore, the probabilistic nature of the stimulus presentation on each trial prevents the animal from predicting which option will be presented in the next trial.

After making their decision, if an option selected led to a reward (as per the programmed reward contingencies associated with each option; Fig. 1), then the unselected option disappeared and the chosen option remained on the screen and a juice reward was delivered. If an option selected led to no reward, then no juice was delivered. The outcome phase lasted for 1.5 s. Each reward was composed of two 0.6-ml drops of blackcurrant Ribena juice (25% blackcurrant Ribena and 75% water) delivered by a spout placed near the animal’s mouth during scanning. Each animal performed up to 200 trials per session.

Each animal underwent two experiments for a total of 20 sessions in each one (four animals × five sessions). Experiment 1 consisted in two separate conditions: One condition used TUS targeted to lOFC area 47/12o. In the other condition no stimulation was applied (sham). Sham and TUS days were interleaved and counterbalanced in order. To ensure that the behavioral effects of TUS were not induced by nonfocal modulation of activity in prefrontal cortex, we ran an additional control experiment (experiment 2), but this time, targeting a separate prefrontal area—aPFC before the animals underwent the same task. Experiment 2 also consisted of two separate conditions: one control condition in which no stimulation was applied (sham) and the active control condition in which TUS was applied to a control area aPFC. “Again, sham and TUS days were interleaved and counterbalanced in order”. In each experiment, each of the four animals performed five sessions, and the order of experiments was counterbalanced across animals (two animals performed experiment 1 first and then experiment 2, and two animals performed experiment 2 before experiment 1). Thus, each animal performed five sessions of the sham condition that were interleaved with five session of the TUS condition (five 47/12o TUS sessions in experiment 1 and five aPFC TUS sessions in experiment 2) in the MRI scanner. Because there were no differences in sham performance in experiments 1 and 2, all sham data were combined in the final analyses. No statistical methods were used to predetermine sample sizes but our sample sizes are similar to those reported in previous publications (*7*, *9*, *11*, *25*). The experiment was controlled by Presentation software (Neurobehavioral Systems Inc., Albany, CA).

### Transcranial focused ultrasound stimulation

A single-element piezoelectric ultrasound transducer (H115-MR, diameter of 64 mm, Sonic Concept) with a 51.74-mm focal depth was used with a coupling cone filled with degassed water and sealed with a latex membrane (Durex). The ultrasound wave frequency was set to the 250-kHz resonance frequency, and 30-ms bursts of ultrasound were generated every 100 ms with a digital function generator (Handyscope HS5, TiePie Engineering). Overall, the stimulation lasted for 40 s. A 75-W amplifier (75A250A, Amplifier Research) was used to deliver the required power to the transducer. A TiePie probe connected to an oscilloscope was used to monitor the voltage delivered. The recorded peak-to-peak voltage was constant throughout the stimulation session. Voltage values per session ranged from 130 to 136 V and corresponded to a peak negative pressure ranging from 1.17 to 1.29 MPa, respectively, as measured in water with an in-house heterodyne interferometer (*39*).

The 40-s TUS train was applied several minutes before each testing session using what has been referred to as an “offline” procedure. This protocol has previously been shown to modulate neural activity for up to 2 hours (*6*, *8*), and the temporal separation between TUS application and task performance makes it difficult to attribute any TUS effects to any possible auditory distraction during the task. Monkeys sat in an MRI-compatible chair in a sphinx position with their heads fixed to the chair through the implanted head post. Each animal’s head was registered to a frameless stereotaxic Brainsight Neuronavigation system (Rogue Research, Montreal, Canada) in advance of the session using a previously acquired T1-weighted image. The anatomical localization of fiducial markers used for each animal was also acquired at the same time as the collection of the T1-weighted image for that monkey. The registration of the fiducial markers to the T1-weighted image of each animal made anatomically precise spatial navigation within the brain of each subject possible, and, therefore, the same exact area could be identified and stimulated in each session. The transducer was positioned using the Brainsight Neuronavigation system so that the focal spot was centered on the targeted brain region. In experiment 1, the targeted brain region was in caudal lOFC area 47/12o [Montreal Neurological Institute (MNI) coordinates *x* = −16, *y* = 8, *z* = −3; *x* = 16, *y* = 8, *z* = −3], in a region anatomically overlapping with the lOFC ROI discussed by Chau and colleagues (*11*). In experiment 1, to ensure complete modulation of activity in 47/12o, bilateral stimulation was applied to the same region in each hemisphere. In the control experiment 2, TUS was instead applied to aPFC (MNI coordinates *x* = 0, *y* = 23, *z* = 11) corresponding to the target previously stimulated in a separate resting-state fMRI experiment (*8*). As in the protocol reported by Verhagen *et al.* (*8*), TUS was applied once for 40 s on the midline through the interhemispheric fissure. As we previous showed (*8*), the TUS parameters used in the current study generated a beam of ultrasonic waves with a 10-mm-wide diameter that targeted aPFC in both hemispheres with a single sonication.

The ultrasound transducer/coupling cone montage was directly positioned on previously shaved skin on which conductive gel (SignaGel Electrode, Parker Laboratories Inc.) had been applied. The coupling cone filled with degassed water and gel applied to the skin was used to ensure ultrasonic coupling between the transducer and the animal’s head. Following stimulation, the monkeys were moved to the MRI scanner for testing. The TUS procedure lasted for 20 min on average.

In each experiment, sham sessions were interleaved with TUS sonication days and completely mirrored a typical stimulation session (the experimental laboratory setting was the same as were the procedures involved in preparing for stimulation such as neuronavigation and cone positioning and application of the cone to the shaved skin on the head of the animal) except that sonication was not triggered.

To test for the specificity of 47/12o TUS, in experiment 2, 20 sham and 20 aPFC TUS (four animals × five sessions) sessions were collected using the same experimental design as in experiment 1. TUS and control days were interleaved in one of two pseudo-random orders that were counterbalanced across animals in each experiment (order 1: S, S, R, T, R, T, R, T, R, T, R, T, R, S, R, S, R, S; order 2: T, T, R, S, R, S, R, S, R, S, R, S, R, T, R, T, R, T) where T, S, and R stand for TUS, sham, and rest days, respectively.

### Statistical analyses

To analyze the behavior of the animals in the three experiments and estimate the effects of no ultrasound stimulation (sham), 47/12o TUS, and aPFC TUS on reward-guided learning and decision-making, we used multiple linear or logistic regressions as implemented in MATLAB. For logistic functions, we used a logit link with categorical predictors. All regressors were normalized to ensure between-session and between-condition commensurability of the regression coefficients. For each session, one β regression weight was extracted for each regressor. These were then tested for statistical significance across all animals/sessions. All data were shown as means ± SEM. Probabilities of *P* < 0.05 were considered as significant.

### Win-stay/lose-shift analyses for behavioral analyses

To estimate the effect of 47/12o TUS and aPFC TUS on behavior, we examined how each animal’s current choice was influenced by choices they had made and outcomes they had received in the prior trial when that choice was last available. We analyzed different strategies using logistic regressions analysis to determine which combination of factors best explained choice:

1) Win-stay/lose-shift strategy: maintenance of the same choice on the next trial (“stay”) after a reward outcome on the current trial (“win”), coded as 1 and shifting to the alternative choice (“shift”) after a no-reward (“lose”) outcome on the current trial coded as −1, this is done separately for each potential stimulus (*A*, *B*, and *C*) and the regressions coefficients are then averaged;

2) Win-stay AND lose-shift strategies: same as previous but encoded as separate regressors. These represent adaptive behaviors;

3) Win-shift AND lose-stay strategies: shifting to the alternative choice (shift) after a reward (win) outcome on the current trial and maintenance of the same choice on the next trial (stay) after a no-reward outcome on the current trial (lose), encoded as separate regressors. These represent maladaptive behaviors.

### Credit assignment matrices

Logistic regression analysis of the animals’ choices used methods identical to those used previously (*22*, *23*, *40*). To determine how recently made choices and recently received outcomes influenced subsequent choices, we conducted three separate logistic regression analyses for *A*, *B*, and *C*, alternatives that the animal could select. All analyses followed the same logic as described here for choice A: We first constructed vectors for choices made to A (coded as 1) and 0 otherwise (choices B and C). We then formed regressors based on all possible combinations of choices and reward from the recent past, trials *n* − 1 to *n* − 4. For each choice-outcome interaction, the regressors were set to 1 when the animal chose A and/or was rewarded and set to 0 otherwise. Nans were used when the choice was not on the screen. A logistic regression was then fit to these 16 explanatory variables (EVs) (i.e., 4 by 4 matrix of all combination of previous choices and outcomes from preceding four trials). Of these, the nine predictors constructed from the three most recent trials were of interest, whereas the remaining seven that involved *n* − 4 trials were included to remove the influence of longer term choice/reward trends.

### Reinforcement model architecture

We fitted the full model specified in our previous work (*2*) on our data. The code for the model can be found at https://osf.io/358cg/?view_only=0e6fda7925364d86930374cd4ae4a59f.

Three value estimates [*Q*(*A*), *Q*(*B*), and *Q*(*C*)] tracked the rewards received for choosing each of the three stimuli that were presented in each session. Note that each session used new stimuli to avoid carry over effects. All *Q* values were initialized at 0.5 at the start of each session, and the chosen stimulus was updated at the time of feedback delivery. As in our previous report, the model entertained a memory trace of reward that was unlinked to specific choices made (R-trace). R-trace was updated on every trial on the basis of the discrepancy between R-trace and the observed outcome, scaled by a learning rate α_R_

$${\text{R-trace}}_{t+1}={\text{R-trace}}_{t}+\alpha_{.R}(r_{t}-{\text{R-trace}}_{t})$$ (1)

Note that the R-trace calculation was independent of the specific choices taken, and hence, only knowledge about the actual sequence of outcomes was required to calculate R-trace. α_R_ was bound between 0 and 1. R-trace was initialized at zero. R-trace influenced *Q* value updates directly. It was inserted directly into the calculation of the prediction error (PE) scaled by a weight parameter *w*_R_, which was allowed to range between −1 and 1. For example, if option *A* was chosen on trial *t*, then its PE and value update for the chosen option would be calculated on the basis of the reward *r* (0 or 1 for reward and no reward) and R-trace as follows

$${\text{PE}}_{t}(A)=r_{t}+w_{.R}{\text{R-trace}}_{t}-Q_{t}(A)$$ (2)

$$Q_{t+1}(A)=Q_{t}(A)+\alpha {\text{PE}}_{t}(A)$$ (3)

α was bound between 0 and 1. We refer to the decision variable (DV) based on these *Q* values as DV_RL_. It reflected the evidence for making a rightward choice. Note that the identity of the left and right choice (whether they were options *A*, *B*, or *C*) was pseudo-random. Below, we explain how the actual DV lastly used was constructed by combining DV_RL_ with a number of choice memory traces: information about the history of stimuli chosen regardless of rewards received [choice stimulus trace (CS-trace)] and the history of locations chosen regardless of the rewards received [choice location trace (CL-trace)]. DV_RL_ also contained a free parameter accounting for a side bias in choice (bound between −1 and 1).

Both choice memory traces were initialized at zero at the start of a session and updated in the following ways. The CL-trace was updated on every trial on the basis of the discrepancy between the actual choice location (*L*), coded as −1 or 1 (for the right and left sides, respectively), and the CL-trace, scaled by a learning rate α_CL_

$${\text{CL-trace}}_{t+1}={\text{CL-trace}}_{t}+\alpha_{.CL}(L_{t}-{\text{CL-trace}}_{t})$$ (4)

The CS-trace decayed exponentially from one trial to the next one with a given rate determined by a free parameter λ_CS_ (*41*, *42*). However, the CS-trace for the chosen option was set to 1 at the end of the trial. For example, the decay of the CS-trace for a stimulus *A* on trial *t* was calculated as

$${\text{CS-trace}}_{t}(A)=\lambda_{CS}{\text{CS-trace}}_{t-1}(A)$$ (5)

CL-trace and CS-trace required only knowledge of the choice location and choice stimulus, respectively, ignoring the sequence of outcomes experienced over the course of a session. λ_CS_ and α_CL_ were bound between 0 and 1.

The choice memory traces exerted their influence on learning and choice scaled by weight parameters. In all cases (also R-trace), the weight parameters could be positive, zero, or negative, meaning that the magnitude and direction of influence of the memory traces were determined empirically during model fitting. CL-trace and CS-trace were added to the DV. The CL-trace could be added directly, because it was already coded in terms of spatial location similarly to the DV itself (although inverted). For the CS-trace, the influence on choice was determined by the difference in CS-trace between right and left stimulus. Note that the CS-trace difference was added to the DV after the trial-wise decay but before the update of the chosen stimulus

$${\text{CL-bonus}}_{t}=-{\text{CL-trace}}_{t}$$ (6)

$${\text{CS-bonus}}_{t}={\text{CS-trace}}_{t}(\text{right})-{\text{CS-trace}}_{t}(\text{left})$$ (7)

$${\text{DV}}_{\text{RL},t}={\text{DV}}_{\text{RL},\text{simple},t}+w_{.\mathrm{CL}}{\text{CL-bonus}}_{t}+w_{.\mathrm{CS}}{\text{CS-bonus}}_{t}$$ (8)

Note that positive values of *w*_CL_ indicate that the DV of the current trial would be biased toward the same location as the direction of previous choices, and positive values of *w*_CS_ indicate that the DV would be swayed toward the stimulus with the highest CS-trace. That is, positive values of *w*_CL_ and *w*_CS_ reflect a tendency to repeat predominant previous choice locations and predominant previous choice stimuli, respectively. R-trace was directly added to the PE calculation as described above.

The final DV was filtered through a standard softmax function to calculate the probability of a rightward choice

$$p_{t}(\text{right})=\frac{1}{1+e^{-\beta \times {\text{DR}}_{\text{RL},t}}}$$ (9)

The inverse temperature parameter β was bound between 0 and positive infinity. The probability of the observed choice on each trial was calculated as

$$p_{t}(\text{choice})=\{\begin{array}{c} p_{t}(\text{right}),\text{if right option is chosen}\\ 1-p_{t}(\text{right}),\text{otherwise} \end{array}$$ (10)

### Model fitting

We used an iterative maximum a posteriori approach previously described (*2*, *43*). This method provides a better estimation than a single-step maximum likelihood estimation (MLE) alone by being less susceptible to the influence of outliers. It does this via implementing two levels: the lower level of the individual subjects and the higher-level reflecting all the data from the different session types, either sham and 47/12o or sham and aPFC, aggregated across all the subjects. In this way, we ensured that the parameters fitted were equally appropriate to both the sham data and the TUS data (either 47/12o or aPFC TUS) when comparisons were made between them. This ensured, for example, that, if there was less choice value related activity after 47/12o TUS in an fMRI analysis, then it could not be explained away as simply a consequence of a poorer model fit to the 47/12o TUS data than to the sham data (*44*). It is for this reason that separate choice value effects and GRS effects are shown for the sham group in Figs. 3, I and J, and 4F. In each case, the sham condition effects have either been estimated when the model included the sham and the 47/12o TUS conditions or sham and aPFC conditions. However, as can be seen, there are only very small differences in the effect estimates derived from either model-fitting procedure.

Briefly, for this procedure, we initialized group-level Gaussians as uninformative priors with means of 0.1 (plus some added noise) and variance of 100. During the expectation, we estimated the model parameters for each subject using an MLE approach calculating the log-likelihood of the subject’s series of choices given the model. We then computed the maximum posterior probability estimate given the observed choices and given the prior computed from the group-level Gaussian and recomputed the Gaussian distribution over parameters during the maximization step. We repeated expectation and maximization steps iteratively until convergence of the posterior likelihood summed over the group or a maximum of 800 steps. Convergence was defined as a change in posterior likelihood of <0.001 from one iteration to the next. Note that bounded free parameters (for example, the learning rates) were transformed from the Gaussian space into the native model space via appropriate link functions to ensure accurate parameter estimation near the bounds.

### MRI data acquisition

Awake animals were head-fixed in a sphinx position in an MRI-compatible chair. fMRI data were collected using a whole-body 3T MRI scanner with a custom-made four-channel phased-array local receive coil in conjunction with a radial transmit coil (Dr. H. Kolster, Windmiller Kolster Scientific, Fresno, CA, USA). Whole-brain blood-oxygen-level-dependent (BOLD) fMRI data were acquired using a gradient echo T2* echo planar imaging (EPI) sequence with 1.5-mm^3^ isotropic voxel size resolution, repetition time (TR) = 2.28 s, echo time (TE) = 30 ms, and flip angle = 90°, and reference images for artifact corrections were also collected. Proton density–weighted images using a gradient-refocused echo (GRE) sequence (TR = 10 ms, TE = 2.52 ms, and flip angle = 25°) were acquired as references for body motion artifact correction. T1-weighted magnetization-prepared rapid gradient echo images (0.5-mm^3^ isotropic voxel size resolution, TR = 2.5 ms, TE = 4.01 ms, inversion pulse time = 1.1 s, and flip angle = 8°) were acquired in separate anesthetized scanning sessions [anesthesia induction via intramuscular injection of ketamine (10 mg/kg), xylazine (0.125 to 0.25 mg/kg), and midazolam (0.1 mg/kg)] before the beginning of the testing sessions.

### MRI data preprocessing

Magnetic resonance images were preprocessed and analyzed using tools from the FMRIB Software Library (FSL) (*45*) and the Magnetic Resonance Comparative Anatomy Toolbox (MrCat; www.neuroecologylab.org). The T1-weighted images were processed in an iterative fashion cycling through a macaque-optimized implementation of FSL’s brain-extraction tool (BET), radio frequency (RF) bias-field correction, and linear and non-linear registration (FLIRT and FNIRT) to the *Macaca mulatta* McLaren template in F99 space as implemented in MrCat. The GRE image was used to carry out the T2* EPI image reconstruction by an offline-SENSE reconstruction method (Dr. Kolster, Windmiller Kolster Scientific, Fresno, CA, USA).

A T1-weighted group template of the four animals included in the study was constructed by cycling through a two-iteration approach of (i) registration to the initial McLaren template in F99 space, (ii) group averaging, and (iii) registration to the new group template. Specifically, tools from Advanced Normalization Tools (ANTs), as implemented in MrCat, were used for the creation of the group template. At each step, the resulting group template was registered to the source template, thus avoiding drift and retaining registration to F99 space. All coordinates reported (in millimeters) refer to F99 space, and results are visualized on the group template.

Despite head fixation during the EPI acquisition, occasional movements of the animals’ limb and body may cause a distortion in the main (B0) magnetic field in a time-varying manner, thereby producing nonlinear motion-related artifacts in the phase-encoding direction (anterior-posterior) on a slice by slice basis, such as ghosting artifacts, misaligned slices, and signal loss. Additional causes of magnetic field distortion and signal loss in nonhuman primate imaging may be induced by magnetic susceptibility differences in tissue, spatially inhomogeneous magnetic field and spatial differences in the proximity of the head from the RF coils. To correct for these artifacts, using a processing pipeline implemented in MrCat, each slice was registered, first linearly and then nonlinearly to a robust reference based on EPI volumes from the same time series with least distortion. This registration and slice alignment processes were performed volume by volume. To avoid overfitting, the degrees of freedom were constrained in several ways: because distortions are the strongest along the phase-encoding direction, here, anterior-posterior, only distortions along this direction were considered; registration was initialized using priors from temporally neighboring slices; low-order solutions were preferred over high-order registration (rigid > affine > nonlinear); nonlinear degrees of freedom were regularized using b-splines. Additional volumes concomitant with any occasional event in the scanning room that may have caused a potential disruption in the main magnetic field were also removed.

Subsequently, the slice-registered average functional image (EPI) was iteratively linearly and nonlinearly registered to the high-resolution structural reference (T1-weighted) of each subject, which was then registered to the group-specific template using FSL and ANTs tools (*45*–*47*). Automatized brain-extraction of the EPI time series was based on brain masks obtained in the high-resolution structural space. Last, to increase signal-to-noise ratio, EPI images were spatially smoothed using a 3-mm kernel (full width at half maximum) and temporally high-pass–filtered (cutoff, 100 s) to remove slow-moving trends. First-level whole-brain analyses (see below) were performed on the motion-corrected low-resolution images in the original acquisition/native space for each session; the statistical maps for each contrast were then transformed from acquisition/native to the group-template standard space with a single resampling by ANTs for group-level analysis.

### Whole-brain fMRI analysis

After transforming each statistical map for each of the four animals from native space to the group-template standard space, group-level statistical analyses of the functional data were performed adopting GLM and tools implemented in FSL (*45*, *47*). Analysis at the group level was performed on the whole-brain using mixed-effects analysis on the basis of FSL’s FMRIB’s Local Analysis of Mixed Effects (FLAME) 1 + 2. As already noted, to increase the number of fMRI sessions available and improve the quality of the analysis, sham sessions from both experiment 1 (*n* = 20; five sessions per animal) and experiment 2 (*n* = 20; five sessions per animal), thereby creating a pool of 40 no-stimulation sham sessions that were contrasted with either 20 47/12o TUS sessions (five sessions per animal) or 20 aPFC TUS sessions. Note that the order of sham, 47/12o TUS, and aPFC TUS sessions was counterbalanced. As a consequence of postpreprocessing abnormalities in the EPI signal, one 47/12o TUS session was excluded, and a final sample of 19 47/12o stimulation sessions was used. The large number of sessions made it possible to estimate robust mixed-effects statistics and model within-subject and between-subjects variances separately, thereby treating each monkey as a random effect. This modeling approach allows consideration of each monkey as if it was randomly sampled from the general population, therefore making it possible to make inferences not only about the sample of individuals tested but also about the population from which the samples were drawn. Nuisance regressors were used to capture additional noise-related variance in the EPI data. These nuisance regressors were intended to (i) identify noisy volumes after the slice alignment preprocessing step and potential signal loss at the volume level, (ii) capture non-linear effects of the motion-related magnetic field distortions, and (iii) detect potential remaining variance in motion-related magnetic field distortions. All analyses were conducted on the whole forebrain but excluded the midbrain and hindbrain where preliminary analyses suggested that there was signal distortion.

### Whole-brain GLMs

All analyses at the group level were performed on the whole-brain using mixed-effects analysis on the basis of FSL’s FLAME 1 + 2, treating subject as random effects. In the GLM comparing sham and 47/12o TUS, the weight of sham and 47/12o TUS sessions were adjusted (sham = 1; lOFC TUS = 2.1) because of the exclusion of one 47/12o session due to postpreprocessing abnormalities in the EPI signal (see above).

All parametric regressors were normalized to ensure between-session, between-subject, and between-condition commensurability of the regression coefficients. For each session, one β regression weight was extracted for each regressor. These were then tested for statistical significance across all animals/sessions. Cluster-based inference was performed in all analyses on the basis of a cluster-defining threshold of *Z* > 2.3 with a Gaussian Random Field (GRF)–corrected threshold of *P* = 0.05. According to this model, the following GLMs were implemented.

#### GLM1—Neural activity related to adaptive behavioral strategies

We aimed to identify neural activity associated with the use of an adaptive behavioral strategy in the sham condition and after TUS of either 47/12o or aPFC. To optimize reward intake, animals should increase the frequency with which they repeat the same choice after it has led to a reward in the past (adaptive win-stay strategy) and they should switch away and not repeat a choice that did not lead to a reward (adaptive lose-shift strategy). At the same time, the animals should avoid switching away from choices that resulted in a reward in the past (maladaptive win-shift strategy) and avoid staying with a choice that did not lead to a reward (maladaptive lose-stay strategy) (fig. S1). We, therefore, used BOLD imaging as measured by fMRI, to compare neural activity associated with the use of adaptive choice strategies (win-stay and lose-shift) as opposed to maladaptive ones (win-shift and lose-stay). We also examined whether this activity was changed after 47/12o TUS or aPFC TUS. A hemodynamic response function (HRF)—a gamma convolution function (mean, 3 s; SD, 1.5 s)—was used to capture the peak of the BOLD signal. This resembled the HRF used in previous macaque fMRI studies (*7*, *9*, *11*).

*GLM1* included an unmodulated decision constant regressor (*DEC*) time-locked to the onset of the decision (when stimuli appeared on screen) with a duration set to the reaction times of each trial. Six parametric regressors, capturing variance in the BOLD signal underlying the encoding of either the win-stay (WS) or lose-shift (LSh) “adaptive” strategies in relation to the choice that would be made on the very next occasion the same choice was offered (WST1 and LShT1; note that the very next occasion on which the choice taken was offered again might not be on the very next trial). In addition to looking at how the most recent experience of reward influenced whether animals stayed with a choice or switched away from it on the next occasion it was offered, we also looked at the impact of reward delivery/nondelivery on the choices taken further in the future on subsequent occasion that the choice was offered and the occasion after that on which it was offered. Thus, we examined win-stay/lose-shift behavior on the subsequent occasion that the same choice was offered again (WST2 and LShT2) and on the occasion after that, when the same option was offered again (WST3 and LShT3). As well as using these six regressors to capture adaptive behavioral strategies, we also used six additional parametric regressors to captured variance in the BOLD signal related to the behavioral maladaptive strategies win-shift (WSh) and lose-stay (LS). As for the regressors encoding adaptive behavioral strategies one, two, or three trials into the future, maladaptive behavioral strategies were also indexed in relation to the choice that would be made on the very next occasion the same choice was offered (WShT1 and LST1), on the subsequent occasion that the same choice was offered again (WShT2 and LST2), or the subsequent occasion after that when the same option was offered again (WShT3 and LST3). The regressors were normalized (mean, 0; SD, 1) and time-locked at the time of feedback. Two unconvolved categorical regressors for leftward (*UNC_left_*) and rightward (*UNC_right_*) responses were time-locked at the time of feedback with a duration of TR (2.28 s) to capture motion-induced distortion in the magnetic field during the response (*7*). One convolved categorical regressors for left minus right responses time-locked at the time of response; this captured variance in neural activity related to responding to one hand or the other. A contrast was used to identify activity related to the adaptive behavioral strategies (win-stay/lose-shift across all three intervals; on the first, second, and third occasions on which the same choice option subsequently appeared) after controlling for the activity related to maladaptive behaviors (win-shift/lose-stay across all three intervals; on the first, second, and third occasions on which the same choice option subsequently appeared)

$$\begin{array}{c} \mathrm{GLM}1=\beta_{1}\mathrm{DEC}+\beta_{2}\text{WST}1+\beta_{3}\text{WST}2+\beta_{4}\text{WST}3+\\ \beta_{5}\text{LShT}1+\beta_{6}\text{LShT}2+\beta_{7}\text{LShT}3+\beta_{8}\text{WShT}1+\\ \beta_{9}\text{WShT}2+\beta_{10}\text{WShT}3+\beta_{11}\text{LST}1+\beta_{12}\text{LST}2+\\ \beta_{13}\text{LST}3+\beta_{14}\mathrm{UN}C_{\text{left}}+\beta_{15}\;\mathrm{UN}C_{\text{right}}+\beta_{16}\;\text{LminusR}+\varepsilon \end{array}$$

#### GLM2—Neural activity related to choice value

The second fMRI analysis (Fig. 4) followed from the first set of behavioral and neural findings that credit assignment and credit assignment–related activity was impaired after 47/12o TUS (Fig. 3). This result suggests a deficit in the animal’s capacity to credit the value of the outcome received after making a decision to the correct choice option, thereby leading to an impairment in the animal’s capacity to update choice value estimates and to use these estimates to guide decisions. Regressors were built using the reinforcement learning model described by Wittman and colleagues (*2*), as summarized above. As in *GLM1*, *GLM2* included an unmodulated decision constant regressor (*DEC*) time-locked to the onset of the decision (when stimuli appeared on screen) with a duration set to the reaction times of each trial. One additional binary regressor with duration equal to a Dirac delta function of 100 ms was time-locked at the time of feedback onset to capture variance in the BOLD signal associated with the outcome, namely, receipt minus nonreceipt of reward (REWminusNOREW). To capture variance explained by distortions in the magnetic field caused by the response action, two unconvolved categorical regressors for leftward (*UNC_left_*) and rightward (*UNC_right_*) responses were time-locked at the time of feedback with a duration corresponding to the TR (2.28 s). In addition, to regress out variance associated with distortions co-occuring with the outcome receipt, two additional unconvolved categorical regressors for receipt of reward (*UNC_reward*) or no reward (*UNC_noreward*) were time-locked at the time of feedback with a duration corresponding to the TR (2.28 s).

All the following regressors were convolved with the HRF, as described above (see the “Reinforcement model architecture” section). A parametric regressor aimed to capture choice location (*c*_Clo_) was normalized and time-locked at the time of response. Three fully parametric regressors were time-locked at the time of decision and were modulated by the expected value of the chosen (*choV*), unchosen (*uncV*), and unpresented (*unpV*) options based on the reinforcement learning model described above (*2*). Further regressors were derived from the same model. Two parametric regressors were intended to capture CL-trace and the choice trace associated with the unpresented option (*unpCT*). They were normalized and time-locked at the time of decision. An additional parametric regressor modeled the comparison between the stimulus choice traces (CS-traces) associated with chosen and unchosen options (*choT-uncT*). It was also time-locked at the time of decision and normalized. Two parametric regressors time-locked at the time of feedback onset and normalized captured variance in the BOLD signal explained by the reward trace (R-trace) in trials in which reward was either received (*rewTreward*) or not received (*rewTnoreward*) for making a choice.

Contrasts were used to identify activity related to (i) the expected value signal associated with all the choice options regardless of their identity and (ii) combined reward trace signal regardless of whether a reward was received or not (illustrated as GRS in Fig. 5)

$$\begin{array}{c} \text{GLM}2=\beta_{1}\mathrm{DEC}+\beta_{2}\;\text{choV}+\beta_{3}\;\text{uncV}+\beta_{4}\;\text{unpV}+\\ \beta_{5}\;\text{choT}-\text{uncT}+\beta_{6}\;\text{unpCT}+\beta_{7}\;\text{locT}+\\ \beta_{8}\;\text{REWminusNOREW}+\beta_{9}\;\mathrm{UNC}\_\text{reward}+\beta_{10}\;\mathrm{UNC}\_\text{noreward}+\\ \beta_{11}\;c_{\text{Clo}}+\beta_{12}\;\text{rewTreward}+\beta_{13}\;\text{rewTnore}w\mathrm{ard}+\\ \beta_{14}\mathrm{UN}C_{\text{left}}+\beta_{15}\;\mathrm{UN}C_{\text{right}}+\varepsilon \end{array}$$

### ROI analyses

Additional analyses were performed on ROIs centered on regions showing the highest significant differences in ventral prefrontal cortex activity between sham and 47/12o TUS sessions. As these analyses rely on coordinates estimated from contrast activation maps resulting from previously mixed-effects whole-brain GLMs, they are here reported primarily for visualization purposes.

ROIs were manually defined as a 1.5-mm-radius spherical ROI on the group-template image in F99 space (*48*) and warped back to each individual session’s EPI image. Activity within the mask was averaged across all voxels and the mean time course was extracted (table S1 and the Supplementary Materials). For GLM1, ROIs were placed in or adjacent to the lateral orbital sulcus in or adjacent to 47/12o (left hemisphere: *x* = −19.5, *y* = 9.5, *z* = −3; right hemisphere: *x* = 11, *y* = 9.5, *z* = 1; left hemisphere: *x* = −11, *y* = 13, *z* = 3.5). For GLM2, the ROI was placed over the ACC/anterior medial frontal cortex (right hemisphere: *x* = 5, *y* = 22, *z* = 14) and IA cortex (left hemisphere: *x* = −18, *y* = 3.5, *z* = −1). Average values of activity within each ROI were extracted from each session. A total number of 40 sham, 19 47/12o sessions, and 20 aPFC sessions were used. Effect sizes for single sessions and averages within each condition (sham, 47/12o TUS, and aPFC TUS) were extracted for visualization as mean and SEM estimates.

### Software availability

FSL can be downloaded from https://fsl.fmrib.ox.ac.uk/fsl/fslwiki. Tools from ANTs, as implemented in MrCat, can be found at www.rbmars.dds.nl/lab/toolbox.html. Figures were made using tools in FSL, Adobe, and Biorender.com.

## Notes

View/request a protocol for this paper from *Bio-protocol*.
