# Supplementary material for: Ultrasound modulation of macaque prefrontal cortex selectively alters credit assignment–related activity and behavior
Source: Sci Adv. 2021 Dec 15;7(51):eabg7700. doi: 10.1126/sciadv.abg7700 (PMC8673758; doi:10.1126/sciadv.abg7700)
Supplement: Supplementary file 1 — Table S1 Figs. S1 to S10 [file sciadv.abg7700_sm.pdf]

Science Advances
AAAS

Supplementary Materials for

Ultrasound modulation of macaque prefrontal cortex selectively alters credit assignment–related activity and behavior

Davide Folloni*, Elsa Fouragnan*, Marco K. Wittmann, Lea Roumazeilles, Lev Tankelevitch, Lennart Verhagen, David Attali, Jean-François Aubry, Jerome Sallet, Matthew F. S. Rushworth

*Corresponding author. Email: davide.folloni@mssm.edu (D.F.); elsa.fouragnan@plymouth.ac.uk (E.F.)

Published 15 December 2021, *Sci. Adv.* **7**, eabg7700 (2021)
DOI: 10.1126/sciadv.abg7700

This PDF file includes:

Table S1
Figs. S1 to S10

Table 1

GLM	Contrast	Brain Structure	Peak Coordinates (in mm) [x, y, z] in SHAM	Z Value in SHAM	Peak Coordinates (in mm) [x, y, z] in SHAM-47/12o TUS	Z Value in SHAM-47/12o TUS	Peak Coordinates (in mm) [x, y, z] in SHAM-aPFC TUS	Z Value in SHAM -aPFC TUS
GLM 1	Adaptive win-stay/lose-shift signal	47/12o and adjacent orbital and ventrolateral PFC	[23.6, 11.1, 1.51]	4.25	[-18.1, 7.55, -4.02]	3.61	/	/
		Amygdala/Anterior Temporal Cortex	[16.6, 7.04, -13.1]	5.09	[-15.6, 7.55, -7.54]	3.95	[-16.6, 4.53, -10.1]	4.16
			[-22.6, 3.52, -8.05]	5.34	[-24.6, 5.53, -2.01]*	3.55	[-23.1, 4.02, -6.04]	3.64
			/	/	[-19.5, 9.5, -3] (sub-peak)	3	/	/
			/	/	[11, 9.5, 1] (sub-peak)	3.3	/	/
			/	/	[-11, 13, 3.5] (sub-peak)	2.2	/	/
			/	/	/	/	[-14.6, 1.01, -10.6]	3.48
GLM 2	Value Signal	47/12o and adjacent orbital and ventrolateral PFC	[21.6, 17.1, 8.05]	5.96	[22.6, 14.6, 9.56]	4.17	/	/
			[-20.1, 17.1, 8.55]	5.79	/	/	/	/
			[21.1, 17.6, 9.57]	5.74	[22.6, 15.1, 8.55]	4	/	/
			[20.6, 15.1, 12.6]	5.74	/	/	/	/
			[1.9, 14.4, -1.4] (sub-peak)	5.1	/	/	/	/
		Anterior Cingulate/Medial Frontal Cortex	[2, 22, 9.8] (sub-peak)	3.67	[5, 22, 14]	3.5	/	/
			/	/	[11.1, 20.1, 16.6]	4.43	/	/
			/	/	[9.05, 20.6, 17.1]	3.98	/	/
		Amygdala/Anterior Temporal Cortex	[21.1, 8.05, -4.53]	6.16	/	/	/	/
	Reward Trace Signal	Anterior Insular Cortex	[-19.1, 5.03, 1.51]	7.81	/	/	/	/
			[20.6, 4.53, 0]	7.79	/	/	/	/
			[-18, 3.5, -1] (sub-peak)	5.8	/	/	/	/

* [-24.6, 5.53, -2.01] in sham – 47/12o is also close to posterior lateral orbitofrontal (47/12o) target region

Supplementary figures

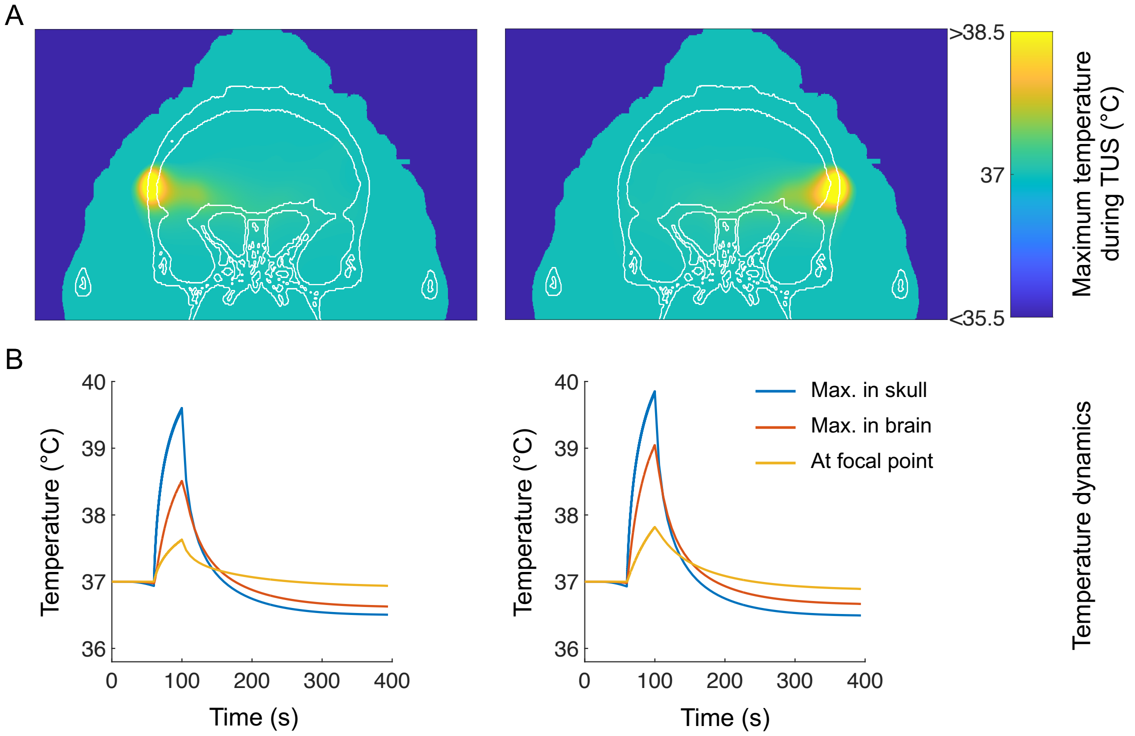

Supplementary Figure 1. Thermal modelling of the offline TUS protocol. (**A**) Whole-head modelling of the maximum thermal rise during 40 s TUS in the right area 47/12o targeting (left column) and left area 47/12o targeting (right column). (**B**) Temperature dynamics for the hottest point in the skull (blue), the hottest point in the brain (red) and the geometrical focal point in the brain (yellow). Given that the skull is more acoustically absorbing than soft tissue, the highest thermal increase was located in the skull itself, estimated by the simulation to be 2.8°C and 2.6°C for left and right, respectively. For an approximate 0.5 mm thickness of the dura the maximum temperature in the brain below the dura was 39.0°C and 38.5°C for left and right, respectively. The maximum temperature at the geometrical focus of the sonic transducer was 37.8°C and 37.6°C for left and right, respectively. As TUS was applied offline, in panel B it can be appreciated how any thermal change in skull, brain or focal point was already back to baseline before the animals was moved into the scanner for the testing session.

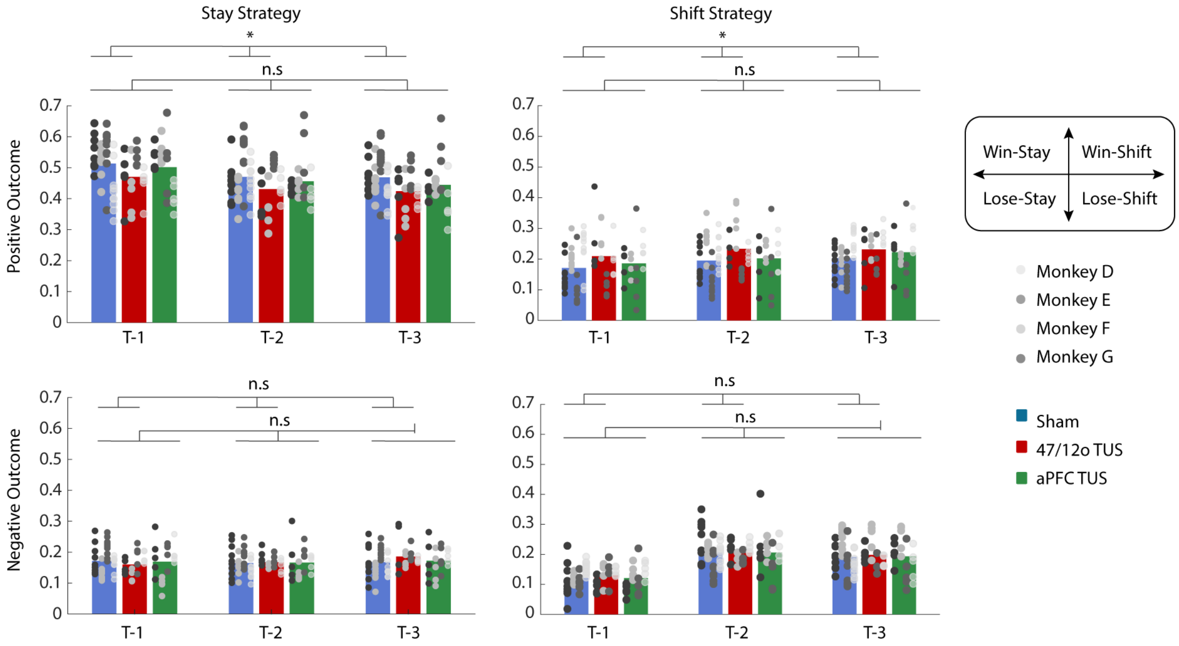

Supplementary Figure 2. Area 47/12o TUS primarily disrupts learning from positive outcomes. (A) In the sham condition the animals tended to follow an adaptive behavioral strategy such as repeating the same choice after receiving a reward for choosing that option on the last occasion it was encountered (win-stay) and they tended to switch away from choices that had led to no reward when they were last chosen (lose-shift). Compared to sham (blue), 47/12o TUS (red) specifically disrupted the ability to make choices based on previously received positive outcomes (top two quadrants) but had a smaller impact on learning from negative outcome (bottom two quadrants). On the contrary, aPFC TUS did not have any impact on the animals' adaptive versus maladaptive behavior.

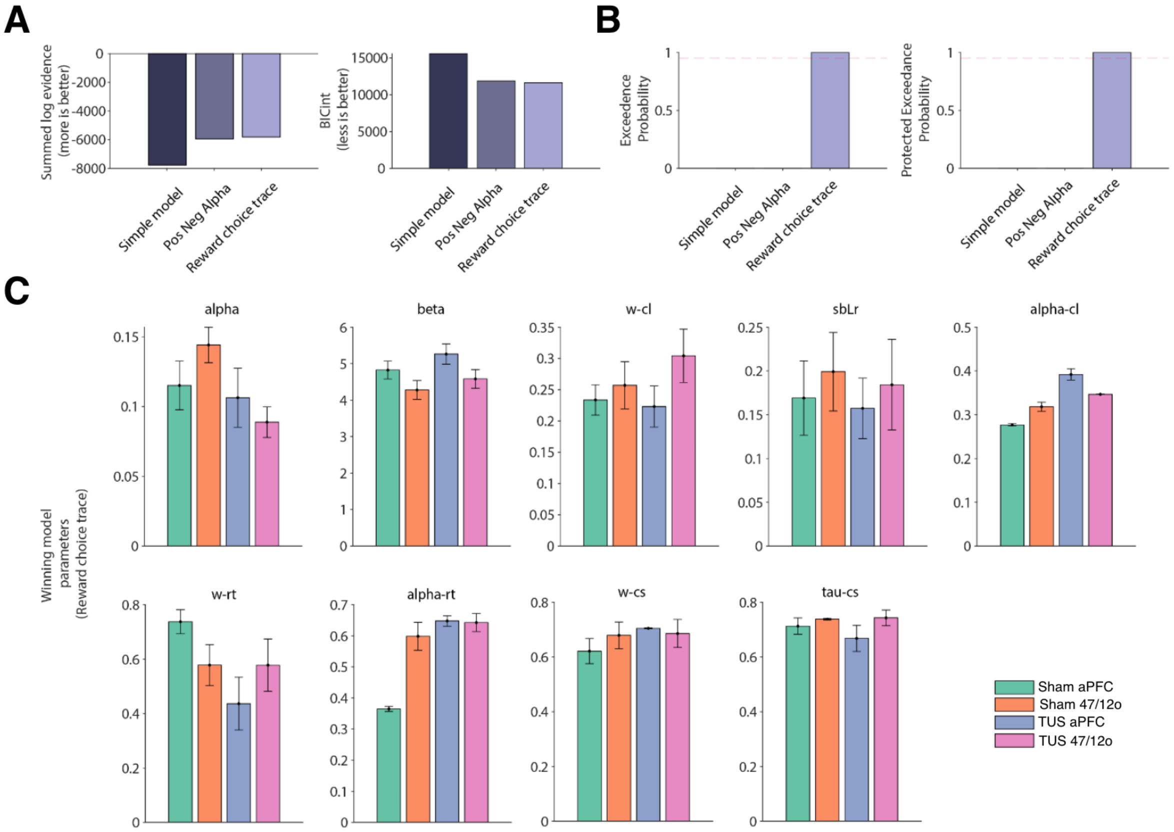

Supplementary Figure 3. (A) Nested model comparison using summed log evidence (more is better) and BICint (lower values indicate better fit). The model including R-trace explains the data best compared to a simple RL model and a model including all parameters and two learning rates for positive and negative outcomes. **(B)** Exceedance probability also favors the R-trace model among our three candidate models. The dashed red line indicates an exceedance probability of 0.95. **C)** All free parameters in the winning model are presented across the four conditions (sham-aPFC, sham-47/12o, TUS-aPFC, TUS-47/12o). From left to right, top panels: learning rate (alpha), exploration parameter (beta), weight parameter for the choice-location (w-cl), side bias (sblr), learning rate for the choice location (alpha-cl), weight parameter for the reward trace (w-rt), learning rate for the reward trace (alpha-rt), weight parameter for the choice-stimulus trace (w-cs), decay of the choice-stimulus trace (tau-cs).

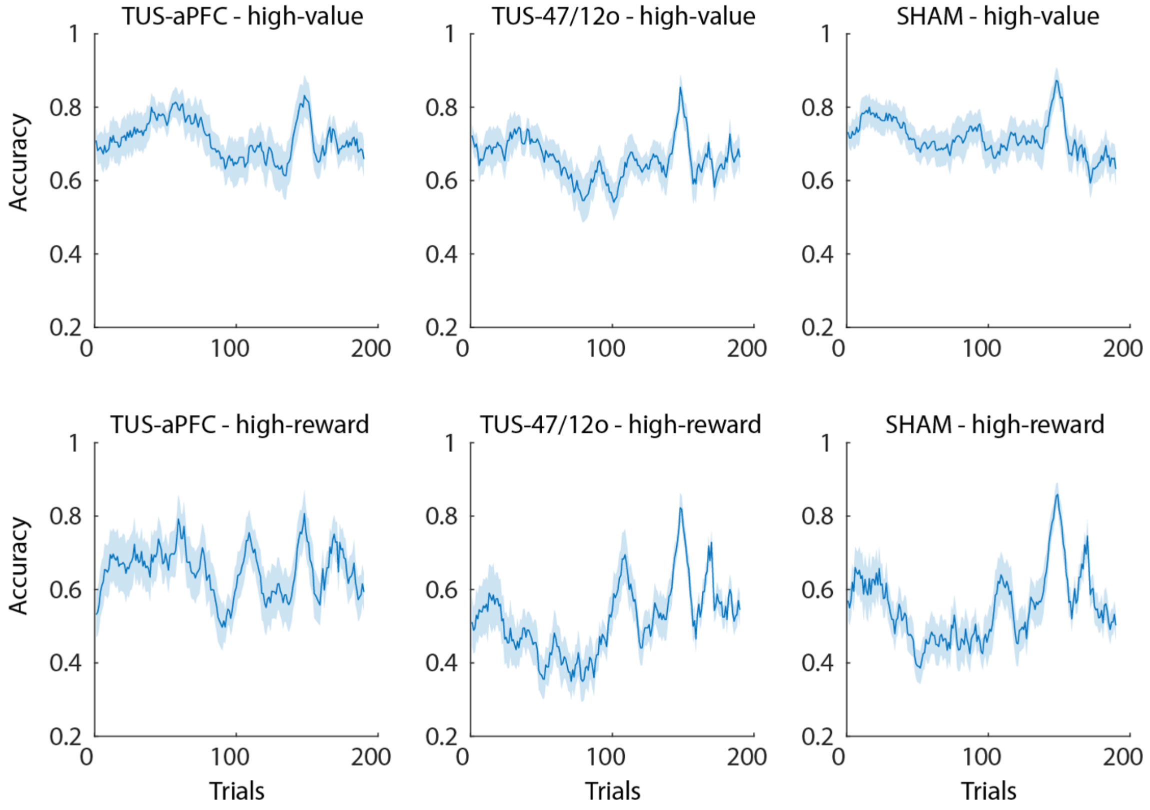

Supplementary Figure 4. Rolling average of the frequency of correct choices. Top panels show the subjective accuracy from the RL model (does the animal select the option with the highest learnt value) across the three experimental conditions. Bottom panels represent the objective accuracy based on reward probabilities associated with each cue across the three experimental conditions.

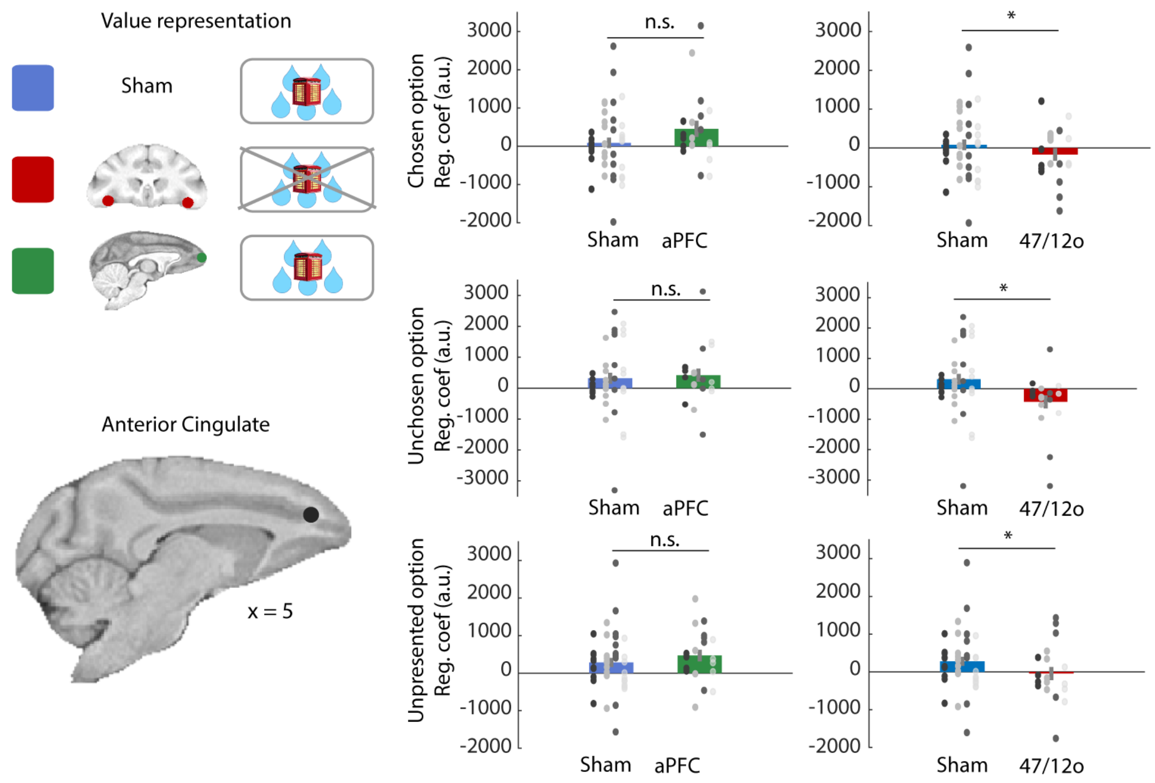

Supplementary Figure 5. Activity in anterior cingulate cortex as a function of choice identity. Activity in anterior cingulate cortex has previously been reported to reflect the value of counterfactual choices, choices that are different to those that are currently being pursued (i.e. the unchosen or the unpresented option as opposed to the chosen option) but which might be chosen on the next occasion they are offered (*11*). The same was true here too in the current experiment; the anterior cingulate cortex activity was positively related to the value of the unchosen and unpresented options (blue bars at the bottom and center) as opposed to the chosen option but these effects changed after 47/12o TUS (red bars) but not after aPFC TUS. As in Figure 3, separate panels illustrate the contrast of sham and 47/12o TUS and sham and aPFC TUS because small differences in model fitting resulted in small differences in effect size estimates in the sham group depending on the comparison group when fitting was done (however careful fitting to either sham versus 47/12o or sham versus aPFC ensured changes in activity were not simply a consequence of a poor model fit when TUS affected behavior).

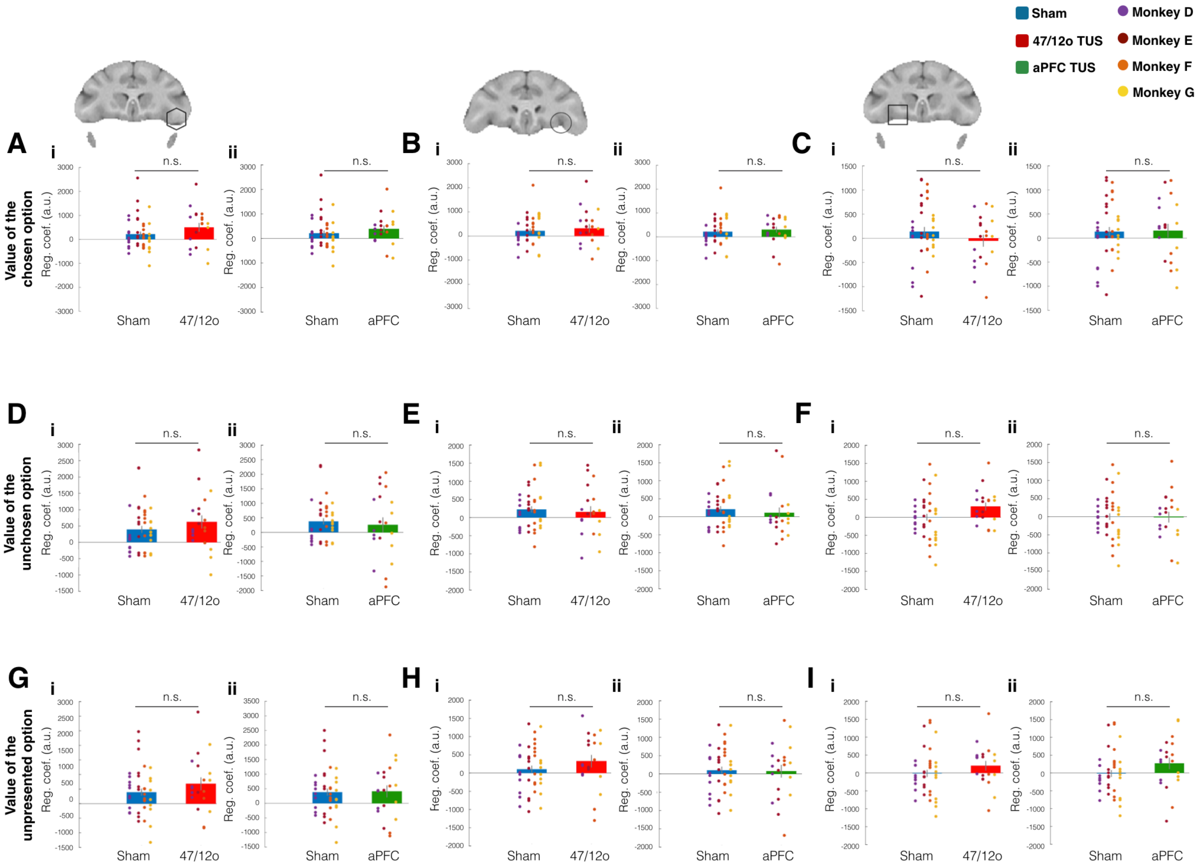

Supplementary Figure 6. Activity in area 47/12o, lateral orbital sulcus and orbitofrontal cortex as a function of value of the chosen, unchosen and unpresented options. Blood oxygen level dependent (BOLD) effects extracted from 1.5mm radius spherical regions of interest (ROIs) centered on regions identified by the comparison of the adaptive behavior effect in the sham and 47/12o condition. The different panels show the extracted BOLD effects associated with the value of the chosen option (A-C), unchosen option (D-F) and unpresented option (G-I). ROIs location was defined on the basis of the results reported in Figure 3C-E and are centered in area 47/12o (A, D, G), lateral orbital sulcus (B, E, H) and adjacent orbitofrontal cortex. Separate panels illustrate the contrast of sham and 47/12o TUS and sham and aPFC TUS because small differences in model fitting resulted in small differences in effect size estimates in the sham group depending on the comparison group when fitting was done (however careful fitting to either sham versus 47/12o or sham versus aPFC ensured changes in activity were not simply a consequence of a poor model fit when TUS affected behavior).

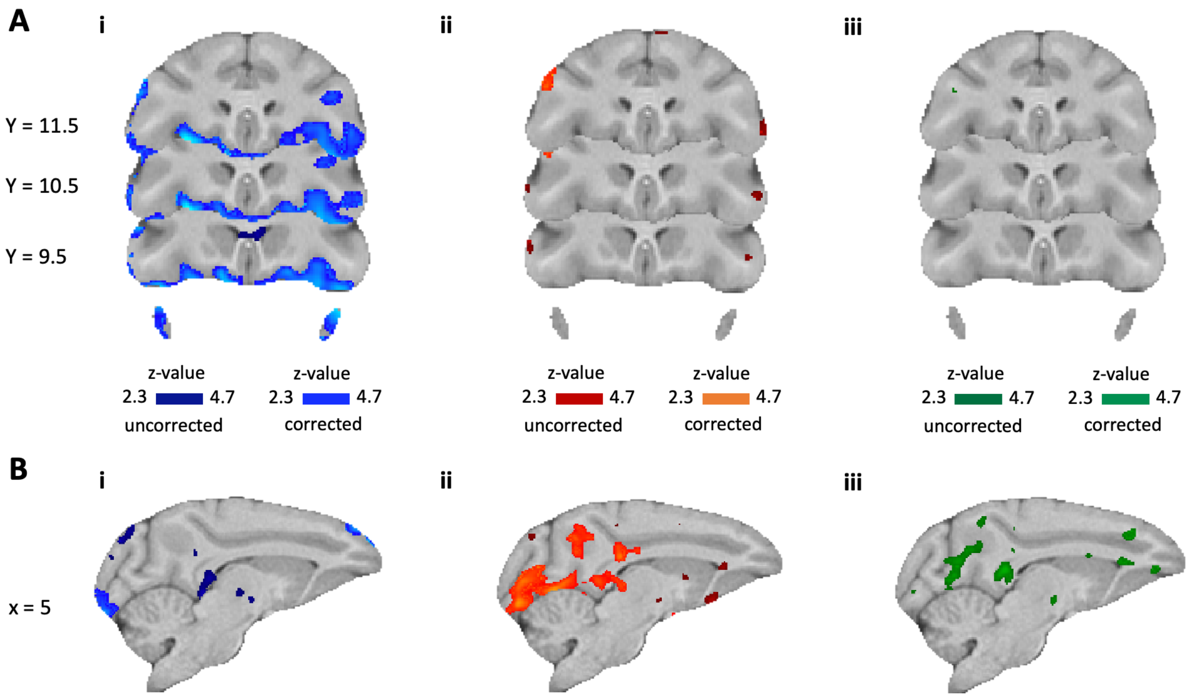

Supplementary Figure 7. Choice value and win-stay/lose-shift activity in ventral and cingulate regions. (A) Neural activity underlying the values of choices was prominent in 47/12o and adjacent orbitofrontal and ventrolateral cortices in the (i) sham condition (cluster corrected $|Z| > 2.3$, $P < 0.05$: light blue). No changes in activity related to the values of choices were apparent in 47/12o and adjacent areas when sham activity was compared after either (ii) 47/12o TUS or (iii) aPFC TUS. Empty brain in i and ii indicates no significant difference for sham versus TUS condition. **(B)** No neural activity related to win-stay/lose-shift was observed in ACC in the (i) sham condition (cluster-corrected, $|Z| > 2.3$, $P < 0.05$) nor was any change in WSLS-related activity observed after either 47/12o TUS (ii) or aPFC TUS. Empty brain in i and ii indicates no significant difference for sham versus TUS condition.

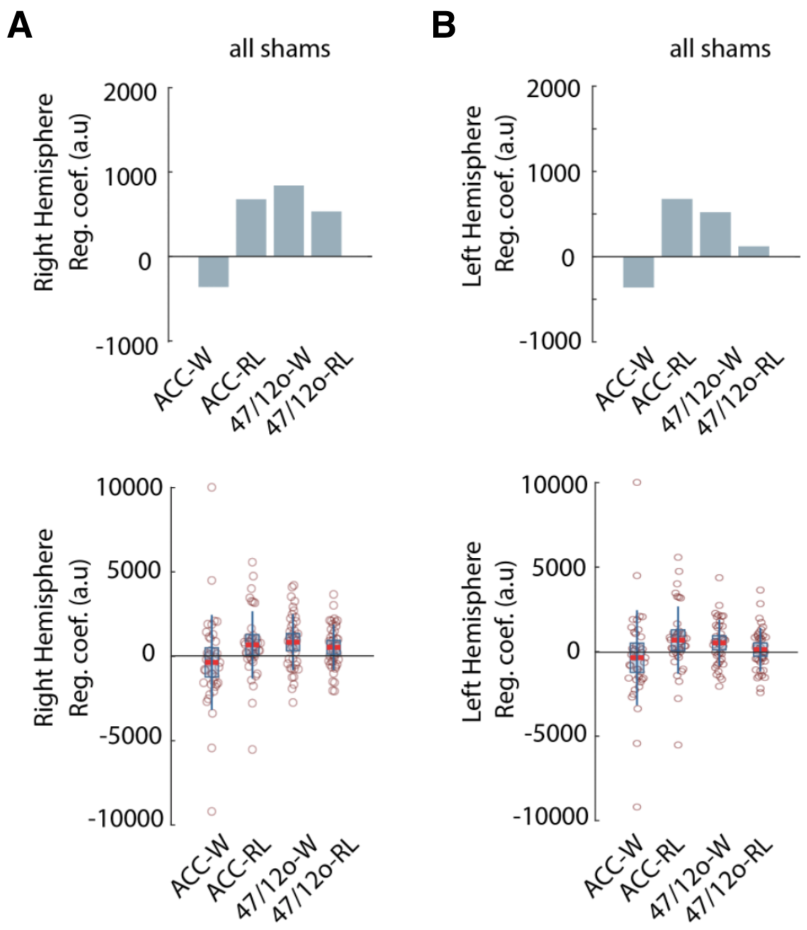

Supplementary Figure 8. We compared the mean regression coefficients in the ACC and the 47/12o areas characterising the relationship between BOLD in these regions and both a WSLS strategy and an RL-derived value representations in the unbiased (SHAM) condition. Top panel shows the mean value, bottom panel shows all sessions betas for transparency. **(A)** Left panels show the activity in the 47/12o ROI in the right hemisphere and **(B)** right panels show the same contrasts for left hemisphere of the same ROI.

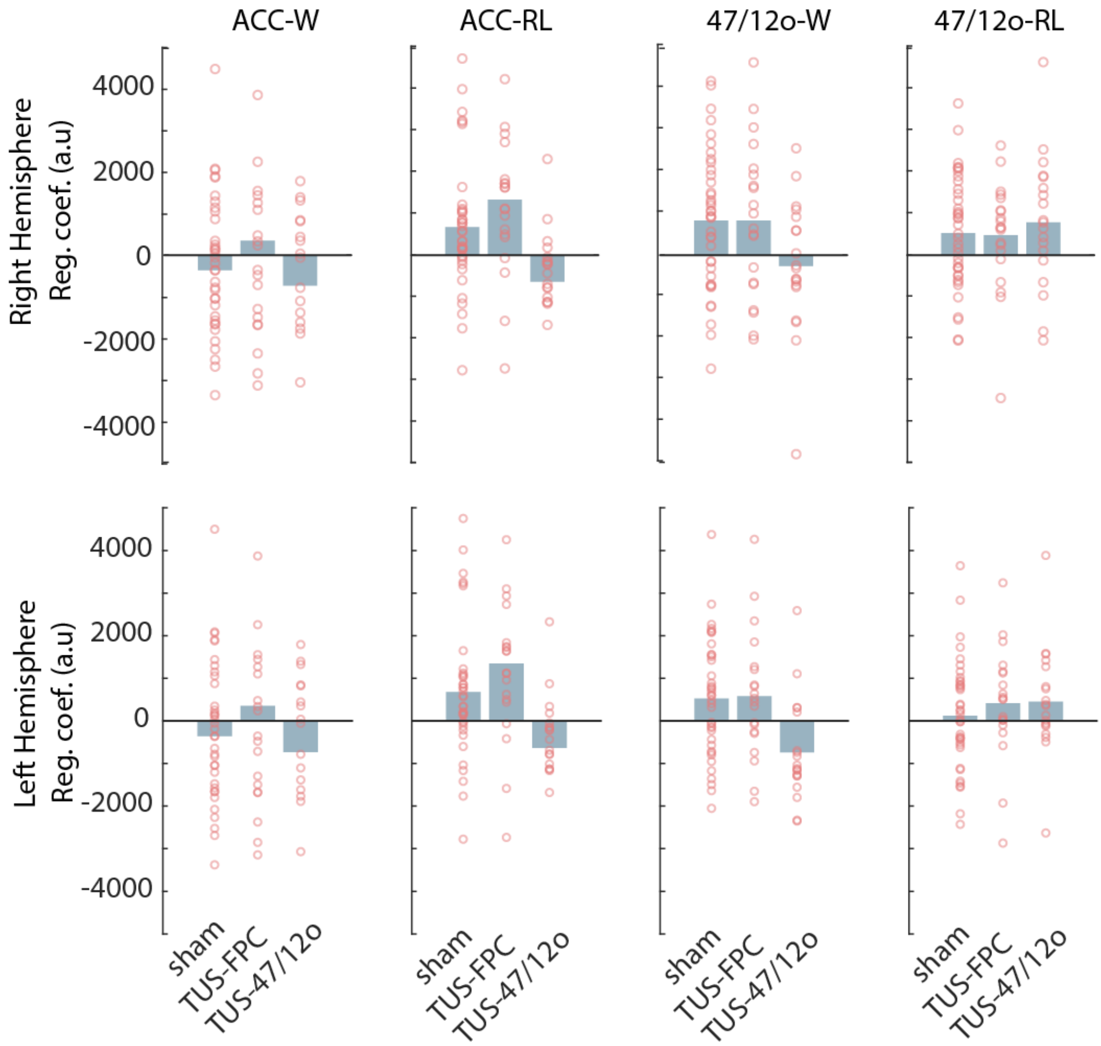

Supplementary Figure 9. Comparison of the relative strengths of value-related activity in or adjacent to area 47/12o in the lateral orbital sulcus and ACC. We directly compared the ACC and 47/12o areas and the two decision strategies (WSLS ad RL) across sham, TUS-FPC, and TUS 47/12o manipulations. Top panels show the activity in the 47/12o right hemisphere and bottom panels show the activity in the 47/12o left hemisphere.

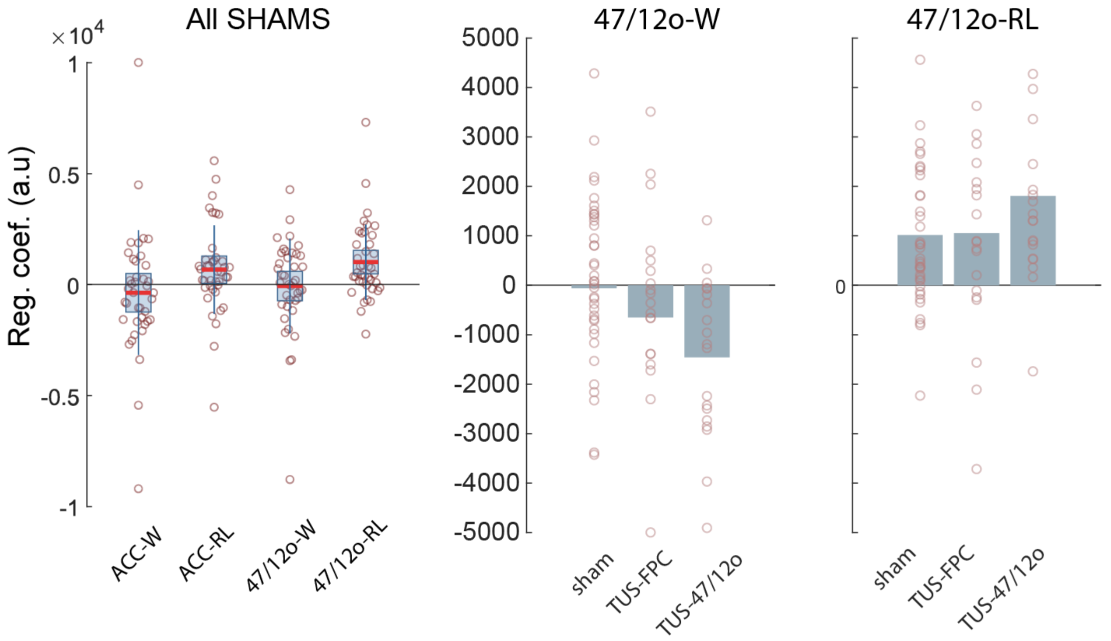

Supplementary Figure 10. Left panel: Comparison of the mean and individual regression coefficients for the more lateral 47/12o ROI (hexagon in Figure 3C-E) and the ACC ROI as a function of different analysis types (values derived from Wittmann's RL model or update-related activity from a WSLS analysis). **Middle and right panels:** Mean and individual regression coefficients in lateral 47/12o ROI (hexagon in Figure 3C-E) for sham, active stimulation (47/12 TUS), control stimulation (aPFC TUS) conditions.
